# Precious metal-free molecular machines for solar thermal energy storage

**DOI:** 10.3762/bjoc.15.106

**Published:** 2019-05-14

**Authors:** Meglena I Kandinska, Snejana M Kitova, Vladimira S Videva, Stanimir S Stoyanov, Stanislava B Yordanova, Stanislav B Baluschev, Silvia E Angelova, Aleksey A Vasilev

**Affiliations:** 1 Faculty of Chemistry and Pharmacy, Sofia University “St. Kliment Ohridski”, 1164 Sofia, Bulgaria; 2 Institute for Optical Materials and Technologies “Acad. J. Malinowski”, Bulgarian Academy of Sciences, 1113 Sofia, Bulgaria; 3 Max Planck Institute for Polymer Research, Ackermannweg 10, 55128 Mainz, Germany,; 4 Institute of Organic Chemistry with Centre of Phytochemistry, Bulgarian Academy of Sciences, 1113 Sofia, Bulgaria

**Keywords:** aza-15-crown-5 ether, benzothiazolium crown ether-containing styryl dyes, *E*/*Z* photoisomerization, molecular solar thermal system

## Abstract

Four benzothiazolium crown ether-containing styryl dyes were prepared through an optimized synthetic procedure. Two of the dyes (**4b** and **4d**) having substituents in the 5-position of the benzothiazole ring are newly synthesized compounds. They demonstrated a higher degree of *trans–cis* photoisomerization and a longer life time of the higher energy forms in comparison with the known analogs. The chemical structures of all dyes in the series were characterized by NMR, UV–vis, IR spectroscopy and elemental analysis. The steady-state photophysical properties of the dyes were elucidated. The stability constants of metal complexes were determined and are in good agreement with the literature data for reference dyes. The temporal evolution of *trans*-to-*cis* isomerization was observed in a real-time regime. The dyes demonstrated a low intrinsic fluorescence of their Ba^2+^ complexes and high yield of *E*/*Z* photoisomerization with lifetimes of the higher energy form longer than 500 seconds. Density functional theory (DFT) calculations at the B3LYP/6-31+G(d,p) level were performed in order to predict the enthalpies (*H*) of the *cis* and *trans* isomers and the storage energies (Δ*H*) for the systems studied.

## Introduction

Molecular photoswitches permanently attract considerable interest because they hold potential for application in molecular electronic and photonic devices [1–5]. Photoswitches are a class of switches that can alternate between the thermodynamically stable forms by application of light (or change of the light intensity) as an external stimulus. If the stable forms (isomers) are not isoenergetic, they have the ability to capture and store imported (solar) energy [6]. As the stored energy is further released as thermal energy, such materials are called molecular solar thermal systems (MOST) [7]. For a pure MOST system the maximal solar energy conversion efficiency was estimated to be 10.6% [8]. It was demonstrated experimentally that by merging a MOST-system [9] with a triplet–triplet annihilation photon energy upconversion system (TTA-UC) [10–11] an effective utilization of sub-bandgap photons is possible [12–13]. Nevertheless, the identification of a precious metal-free MOST system utilizing the visible part of the sun spectrum remains a considerable challenge.

The stated requirements for such systems can be summarized as follows [7]:

(i) the potential barrier between the higher energy form and the lower energy form of the energy carriers should be as high as possible;

(ii) the higher energy form must be stable for a long time;

(iii) the higher energy form has to be with quite small molar absorptivity in comparison to the lower one;

(iv) the quantum yield of the photoisomerization has to be as high as possible, which requires the design of MOST systems with completely suppressed or minimal fluorescence, intramolecular charge transfer or other processes, quenching the photoisomerization;

(v) the energy storing MOST materials have to utilize light in the visible range of the spectrum;

(vi) it will be a crucial benefit for the MOST technology, if toxic and precious metals are avoided. Focusing on environmentally friendly MOST system is an unavoidable requirement;

(vii) the MOST materials should be of low cost and easily accessible; their synthesis and purification should be straightforward, reliable, fast, inexpensive and ideally environmentally benign.

Therefore, only MOST systems fulfilling all requirements (i)–(vii) simultaneously can be regarded as realistic technological systems for long-term solar energy storage.

However, the design and preparation of molecules matching all the above-mentioned criteria is quite difficult. Thus, the invention of new materials possessing at least parts of these requirements provides the base for further improvements and brings the scientists nearer to the identification of the “perfect” MOST system. In this connection we identified crown ether-containing styryl dyes [14–15] as promising substances due to their ability to undergo *trans-*to-*cis* photoisomerization (*E*/*Z*) with very high quantum yields. In addition, their structures predispose to an easy functionalization and low cost synthesis. Another advantage of these dyes is the very low molar absorptivity [16–17] of the higher energy *cis* isomer, which is a prerequisite for its photostability. The *cis* isomers of styryl dyes linked to crown ethers have been identified to form complexes with alkaline-earth metal cations with higher stability constant than the respective *trans* isomer forms [14–15,18]. Lednev et al. proposed as an explanation of the difference in the determined stability constants values for the complexes with *cis/trans* isomers the formation of an additional intramolecular coordination bond between the “crowned” cation and the alkylsulfonate anchoring group which is only possible for the *cis* form [19]. The further studies by the same group of authors indicated that benzothiazolium styryl monoazacrown ether dyes have several main advantages. First of all, the azacrown nitrogen atom is linked directly to the dye and it is part of the chromophore system, responsible for the “push–pull” effect and the photophysical properties of the dye. This provides control over the “push–pull” effect in the chromophore by switching on and off states (i.e., metal-in/metal-out from the crown ligand) [20]. Further, it was found that the azacrown benzothiazolium styryl dyes **4a** and **4c** exhibit ion-sensitivity in the thermal *cis*–*trans* photoisomerization [18,21], a feature, which may be used in the development of thermoreversible photoionic molecular devices [19]. Crown ether-containing styryl dyes are used as sensors for dications such as Ba^2+^, Ca^2+^ and Mg^2+^, or as materials for optical data storage [19]. Thus, to the best of our knowledge this kind of dyes has not been specified as potential MOST material yet.

The aim of the present study is to reveal the potential of the aza-15-crown-5-containing styryl dyes as efficient material for the TTA-UC accelerated MOST process.

## Results and Discussion

### Synthesis

**Scheme 1:**
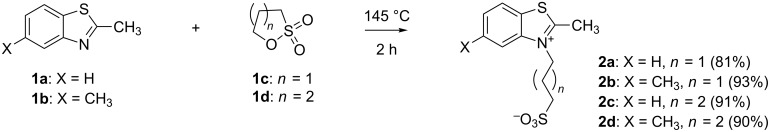
Quaternization of 2-methylbenzothiazoles with alkane sultones.

The synthetic pathway starts with the quaternization of 2-methylbenzothiazoles **1a** and **1b** with 1,3-propanesultone (**1c**) or 1,4-butansultone (**1b**) in a sealed tube at 145 °C without any solvent, as it was described in the literature [17] (Scheme 1).

**Scheme 2:**
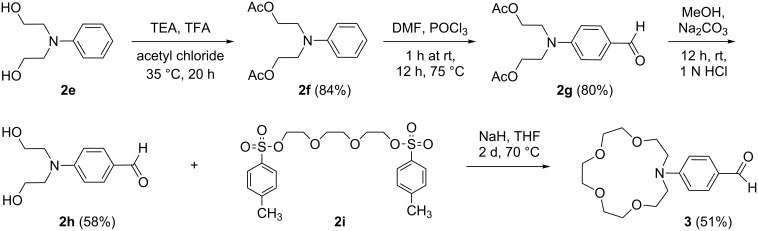
Synthesis of 4-(aza-15-crown-5)benzocarbaldehyde (**3**) [22].

A four-step synthetic pathway was used for the synthesis of 4-(aza-15-crown-5)benzocarbaldehyde **3** (Scheme 2) following the procedure described in [22].

**Scheme 3:**
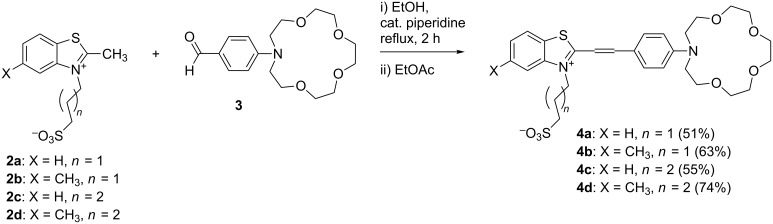
Synthesis of dyes **4a–d**.

A series of the target dyes **4a–d** was prepared using a modified literature procedure (Scheme 3) [22]. The modification consists in the addition of a 10% molar excess of 4-(aza-15-crown-5)benzocarbaldehyde (**3**, Scheme 3) and usage of ethyl acetate as an additional solvent for product precipitation. Because of its better solubility in ethyl acetate the unreacted excess of crown ether **3** was easily removed. In general the zwitterionic salts **2a–d** reacted to complete depletion in ethanol and in the presence of piperidine as catalyst with the crown ether benzaldehyde **3** (TLC monitoring, ethyl acetate/ethanol 4.5:0.5). After the addition of ethyl acetate the precipitated target products **4a–d** were isolated by filtration.

It must be mentioned explicitly, that the modification of the procedure helped us to avoid cost-intensive and time-consuming purification procedures which makes the synthesis suitable for large material quantities. In this way only one further precipitation from ethanol/ethyl acetate 1:3 was needed to obtain analytically pure target dyes **4a–d**.

The dyes **4a** and **4c** were previously described [18,21] and were used as reference compounds. To the best of our knowledge dyes **4b** and **4d** are new compounds. The chemical structures of all dyes from the series were proved by NMR spectroscopy, elemental analysis, IR and UV–vis spectroscopy.

### Photophysical properties

#### Steady-state absorption and emission spectroscopy

**Figure 1:**
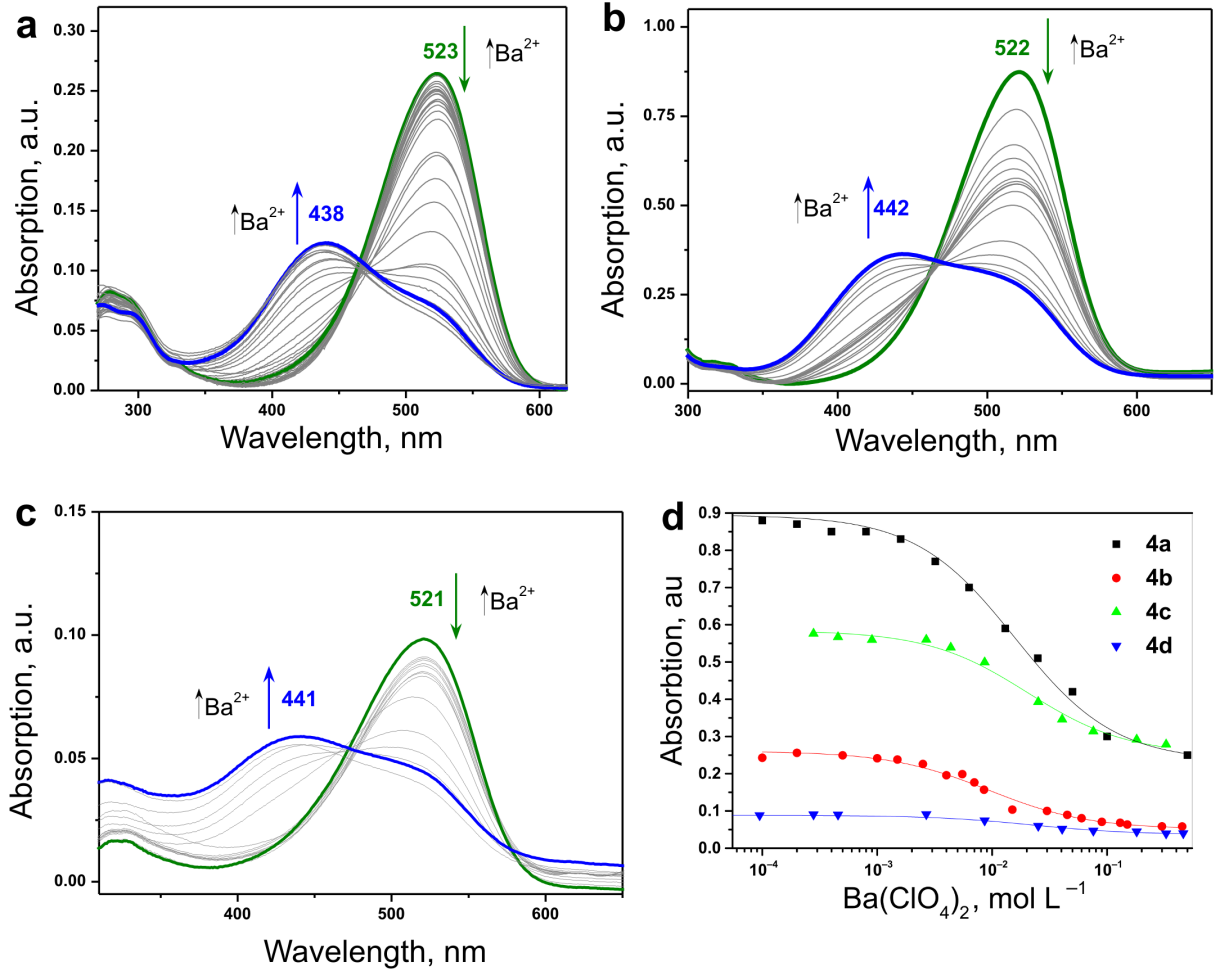
a–c) Dependence of the absorption spectra of the dyes **4b**, **4c** and **4d**, respectively (*c*_L_ = 1.0 × 10^−5^ M in ACN) on the concentration of Ba(ClO_4_)_2_, ranging from 1 × 10^−4^ M up to 5 × 10^−1^ M. d) Absorption of dyes **4a–d** at λ = 520 nm as a function of Ba(ClO_4_)_2_ concentration. Experimental conditions: rt, sample thickness *d* = 10 mm.

Next we elucidated the photophysical properties of the chosen compounds and determined their suitability to be used as MOST systems. First, we defined their photophysical behavior in neat acetonitrile (ACN) solution and in the presence of barium cations. Figure 1 shows the absorption spectra of dyes **4a–d** measured at different concentrations *(c*_M_*)* of Ba(ClO_4_)_2_. All dye solutions have similar absorption profiles with a broad long wavelength band in the vis region (Δλ = 400–600 nm) with long wavelength maxima at λ_max_ = 520 nm indicating that their spectral properties are determined principally by the core chromophore structure. The addition of Ba^2+^ ions to the ACN solution of all dyes induced a decrease in the absorption maximum intensity (at about 520 nm) and to a substantial hypsochromic shift of the maximum absorption with peak at around λ_max_ = 440 nm. The changes in the absorption spectra of the dyes upon the addition of Ba(ClO_4_)_2_ in ACN solution are characteristic for an ion complexation by the azacrown ether group of the chromoionophore and the spectra in Figure 1 were assigned to the formation of the *trans*-dye–Ba^2+^ complex [19]. For all dye solutions, a distinct isosbestic point upon titration was observed, indicating only one kind of complex formation even at the highest Ba^2+^ concentration.

To determine the optimal Ba^2+^ concentration for our measurements it was necessary to define the stability constants for each dye–Ba^2+^ complex. The dependencies of the absorption *A* (Equation 1) of the dyes **4a–d** at a fixed wavelength λ = 520 nm on the Ba^2+^ concentration in ACN is shown in Figure 1d. The curves were approximated by Equation 1, which is true for the simplest form of complexation [18]:

[1] $$A=[A_{0}+A_{\infty }Kc_{\text{M}}]/[1+Kc_{\text{M}}]$$

[2] L+M→LM

where *A*_0_ and *A*_∞_ are the absorptions of the chromoionophore at zero and infinite concentration of the metal ion, respectively; *A* is the absorption at the concentration *c*_M_ of the metal ion; *K* is the stability constant of complex formation, and *L* and *LM* are the ligand and the metal ion complex, respectively. *A*_∞_ and *K* were found by approximation of Equation 1.

The stability constant for the complex formation was estimated to be *K* = 100 ± 15 M^−1^, 49.4 ± 7.6 M^−1^ and 44.7 ± 10 M^−1^ for dyes **4b**, **4c** and **4d**, respectively. For dye **4a** the stability constant was found to be *K* = 70 ± 15 M^−1^ which is in good correlation with the published data [22]. These results prompted us to identify the optimal Ba^2+^ concentration, necessary for a maximum degree of dye–Ba^2+^ inclusion complex formation. Obviously for a better complexation it is necessary to work with high Ba^2+^ concentrations. The explanation is related to the size of the barium cation which does not intercalate completely in the crown ether cavity. This assumption is confirmed by the slight downfield shift of the signals in the ^1^H NMR spectrum of dye **4b** in the presence of Ba^2+^ cations compared to that in neat CD_3_CN (Supporting Information File 1, Figure S9 and Figure S10).

**Figure 2:**
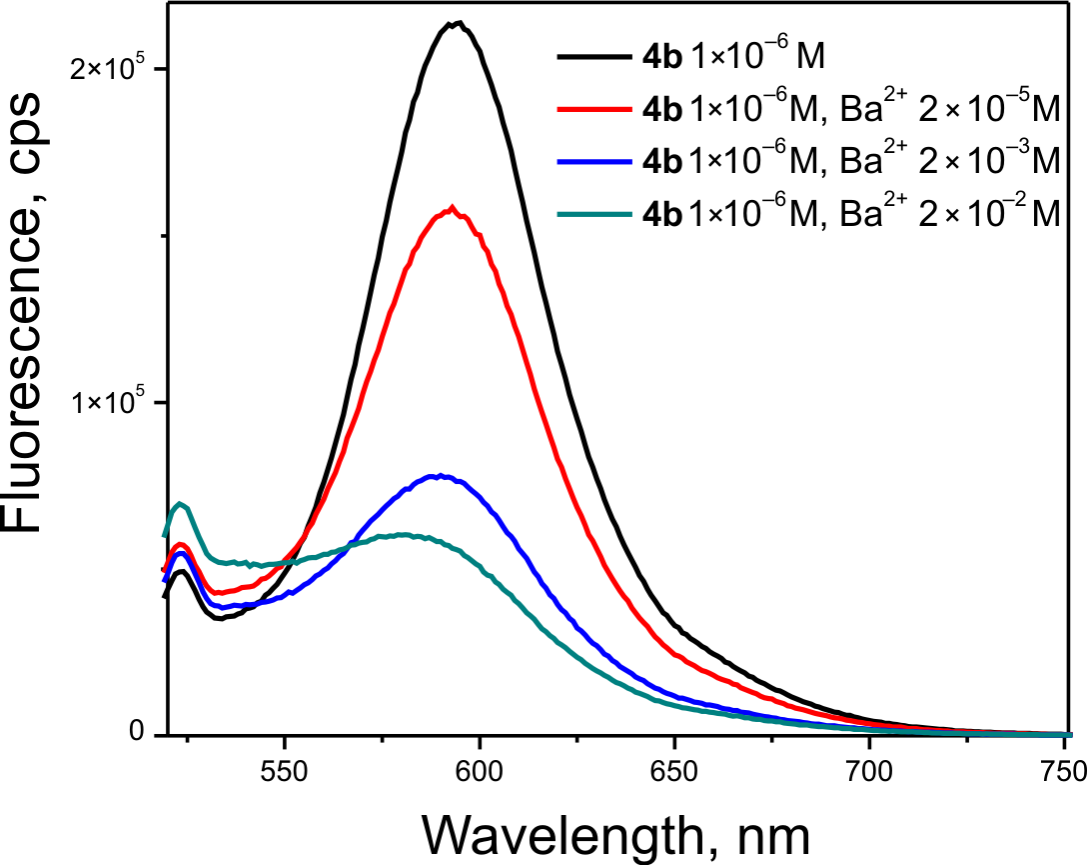
Dependence of the fluorescence of **4b** on the concentration of the Ba^2+^ ions. Excitation wavelength λ_exc_ = 488 nm.

A common behavior demonstrated in Figure 2 was observed: the fluorescence quantum yield of all dyes (Table 1) decreases if the concentration of the Ba^2+^ ions increases, i.e., the formation of the *trans*-dye–Ba^2+^ complexes causes the observed fluorescence decrease.

The optical parameters of all 4 dyes, including absorption maxima (λ_max,abs_), fluorescence emission maxima (λ*_max,f_*), hypsochromic shift of the absorption maxima (Δλ_max_), and fluorescence quantum yields (Φ_f_) for different excitation wavelengths, obtained for the dyes in ACN solution at different Ba^2^*^+^* concentrations are summarized in Table 1. The emission maximum of all dyes is at about λ_max,f_ = 595 nm and does not change significantly upon Ba^2+^ ion complexation. No changes in the shape of the fluorescence curves were observed, when the excitation wavelength was tuned within the region of λ_exc_ = 400–550 nm.

**Table 1:** Absorption maxima (λ_max,abs_), fluorescence emission maxima (λ_max,f_), shift of the absorption maxima (Δλ_max_), and quantum yields of fluorescence (Φ_f_) in % (at 488 nm and 440 nm excitation) for the *trans* isomers of dyes **4a–d** in acetonitrile solution at different Ba^2+^ concentrations.

Dye	*c*_M_/*c*_L_	λ_max,abs_, nm	Δλ_abs_	λ_max,f_, nm	Φ_f_ % λ_exc_ = 488 nm	Φ_f_ % λ_exc_ = 440 nm

**4a**	0	520		597.6	5.9	1.9
	1000	439	81	595	0.29	0.29
	10000	439	81	595	0.34	0.37
**4b**	0	523		594	6.42	0.83
	2000	438	85	588	1.9	0.38
	10000	438	85	580.6	0.91	0.44
**4c**	0	522		593.8	0.23	0.4
	1000	442	80	596	0.18	0.25
	10000	442	80	596	0.14	0.13
**4d**	0	521		599	0.56	0.77
	1000	441	80	596	0.31	0.32
	10000	441	80	594	0.49	0.58

Generally one of the advantages of the presented Ba^2+^–crown ether containing styryl dye complexes as a MOST material is the extremely low intrinsic fluorescence which can be a precondition for higher quantum yields of the *cis–trans* photoisomerization reactions.

#### Real-time E/Z-photoisomerization of dyes **4a–d** and their complexes

**Figure 3:**
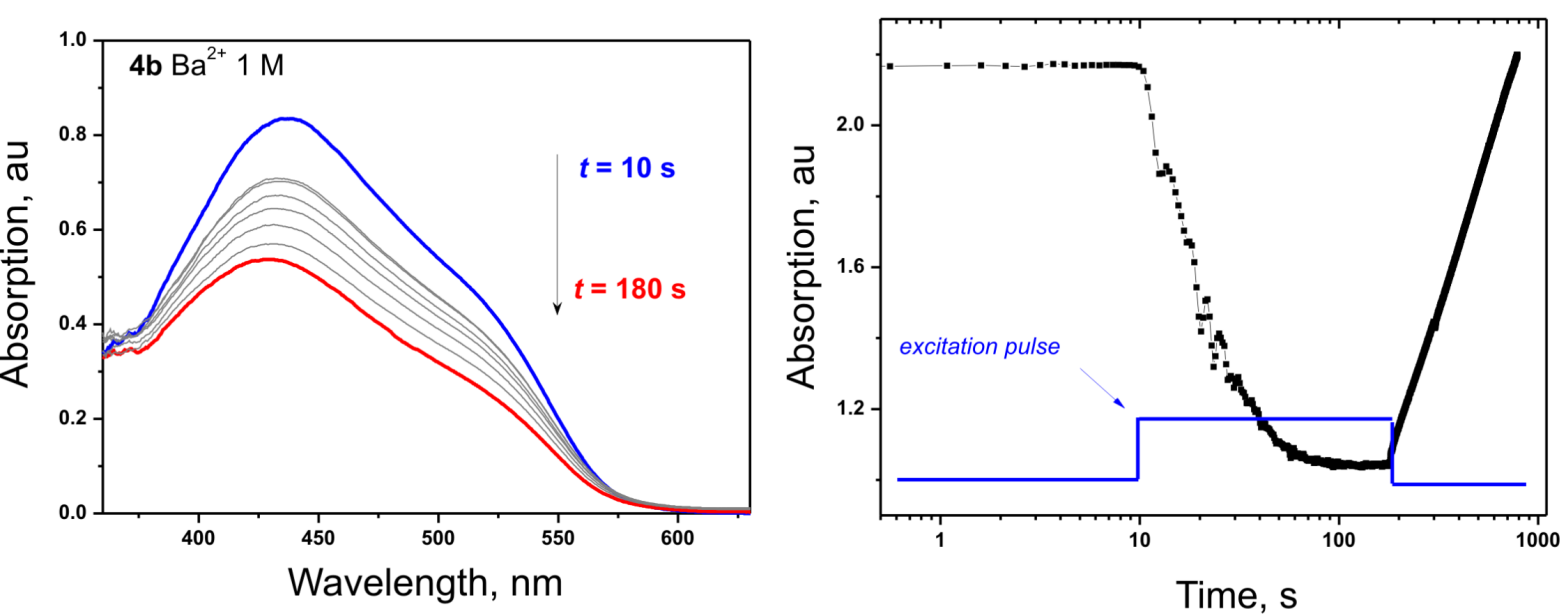
a) Dependence of absorption spectra of dye **4b** (*c*_L_ = 1.0 × 10^−4^ M) with Ba^2+^ (*c*_M_ = 1 M) on the irradiation time. b) Temporal absorption evolution, measured at λ_abs_ = 500 nm, upon irradiation. Experimental conditions: Excitation wavelength λ_exc_ = 488 nm; excitation intensity 14 mW cm^−2^, solvent, ACN; pulse duration *t* = 180 s.

The photoisomerization of free dyes **4a–d** and dye–Ba^2+^ inclusion complexes were investigated in real time mode upon irradiation with visible light (λ = 488 nm) close to their absorption maxima. Figure 3a illustrates the characteristic changes in the absorption spectra of dye **4b**–Ba^2+^ complex under optical excitation with λ = 488 nm and intensity of 14 mW cm^−2^. The temporal evolution of the absorption at specific wavelength of λ_abs_ = 500 nm is demonstrated in Figure 3b: during the first time interval from *t*_1_ = 10 s up to *t*_2_ = 180 s the absorption is decreasing monotonically, as a result of π → π transition in the *trans* isomer of the free dye and its Ba^2+^ complex. During the next time interval for *t*_3_ > 180 s, the optical excitation was terminated and constant increase of the optical absorption was observed.

The degree (*R*) of *trans-*to*-cis* photoisomerization at the photostationary state was evaluated from Equation 3:

[3] $$R=(A_{0}-A_{\infty })/A_{0}$$

where *A*_0_ is the absorption before irradiation and *A*_∞_ is the absorption at the photostationary state. It was found that the rate of the photoisomerization process and the degree of conversion *trans*-to-*cis* isomer depends strongly on the Ba^2+^ concentration. The *cis* isomers formed upon irradiation are thermally unstable and revert to the *trans* isomers in the dark [23]. The rates of *trans*-to-*cis* isomerization were determined for several Ba^2+^ concentrations in the range of 2 × 10^−3^ M up to 1 M at a fixed dye concentration (1 × 10^−4^ M) for all dyes. The kinetic data were found to fit well to a single exponential function (Equation 1), giving a rate constant (*k*) corresponding to a lifetime (1/*k*) [18]:

[4] $$A=A_{\infty }+(A_{0}-A_{\infty })\exp(-kt)$$

where *A*_0_ and *A*_∞_, are the initial and final absorptions, *A* is the absorption at 500 nm at a time *t* after termination of the irradiation. In all cases, after irradiation ceased, complete reversion toward the initial absorption spectrum was observed. As a rule, the reversion from the *cis* to the *trans* isomer leads to a gradual increase in the intensity of the absorption maximum. The photoisomerization data for all 4 dyes are summarized in Table 2.

**Table 2:** Degree (*R*) of *trans-to-cis* photoisomerization at the photostationary state, rate constants (*k*) and lifetime of *trans-to-cis* isomerization.

Dye M	Ba(ClO_4_)_2_ M	*R*	Lifetime s	*k* s^−1^

**4a** 1.0 × 10^−4^	0	0.19	31	32.6 × 10^−3^
	2.0 × 10^−3^	0.53	69	14.4 × 10^−3^
	2.0 × 10^−2^	0.52	264	3.8 × 10^−3^
	2.0 × 10^−1^	0.56	284	3.5 × 10^−3^
	1	0.53	342	2.9 × 10^−3^
**4b** 1.0 × 10^−4^	0	0.35	148	6.0 × 10^−3^
	2.0 × 10^−3^	0.82	292	3.4 × 10^−3^
	2.0 × 10^−2^	0.74	279	3.6 × 10^−3^
	2.0 × 10^−1^	0.54	481	2.1 × 10^−3^
	1	0.39	514	1.9 × 10^−3^
**4c** 1.0 × 10^−4^	0	0.00	0	0
	2.0 × 10^−3^	0.00	0	0
	2.0 × 10^−2^	0.08	0	0
	2.0 × 10^−1^	0.52	330	3.0 × 10^−3^
	1	0.44	390	2.6 × 10^−3^
**4d** 1.0 × 10^−4^	0	0.21	18	55.0 × 10^−3^
	2.0 × 10^−3^	0.23	20.9	47 × 10^−3^
	2.0 × 10^−2^	0.22	30.7	32 × 10^−3^
	2.0 × 10^−1^	0.58	137	7.3 × 10^−3^
	1	0.26	301	3.3 × 10^−3^

Generally, the addition of Ba^2+^ ions was found to increase substantially the lifetime of the *cis* isomers. Under the abovementioned conditions the *cis*-**4b**–Ba^2+^ complex was detected to be most stable, while dye **4d** formed stable *cis-***4d**–Ba^2+^ complex only at a higher concentration of Ba^2+^ ions (1 M).

**Figure 4:**
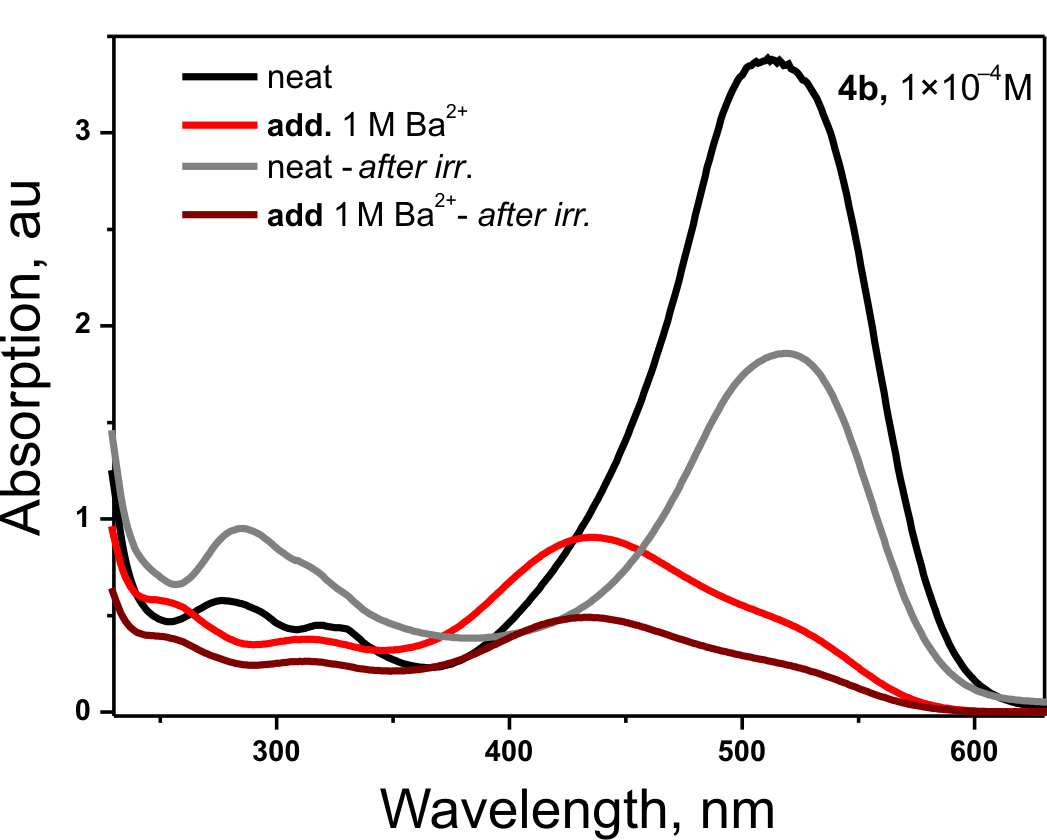
UV–vis absorption spectra of dye **4b** (1.0 × 10^−4^ M) free and in complex with Ba^2+^ (1 M) before and after the end of the exposure at λ = 488 nm.

Figure 4 is an additional demonstration of the ability of dye **4b** to undergo *trans-*to-*cis* photoisomerization in its free form and in complex with Ba^2+^. The absorption band corresponding to the π → π transition in the *cis* isomer (at around 280 nm and 320 nm, shown in Figure 4), as for the free dye **4b**, increased with the irradiation time, suggesting that isomerization from *trans* to a *cis* form of the free dye or its Ba^2+^ complex proceeded until a photostationary state was reached. As can be seen from Figure 4 the *trans-to-cis* isomerization takes place to a higher extent in the free form of the dye than in the complex. However, the lifetimes of the free *cis* form is extremely short in comparison to that of the complex. The degree of photoisomerization of the new dye **4b** is apparently higher than that of the known structure (**4a**). From one side it can be supposed that dye **4a** aggregated much faster than its methyl-substituted analogue **4b**. From another hand the substituent in the 5-position of the benzothiazole heterocycle sterically hinders the rotation of the alkylsulfo-anchoring group and thus plays the role of a controller with regard to its direction towards the crown ether. This finding is a resemblance to the results from reference [24] where the 5-methoxy-substituted benzothiazole styryl-crown ethers demonstrated higher quantum yields of *trans-*to-*cis* photoisomerization.

#### Insight from electronic structure calculations

**Figure 5:**
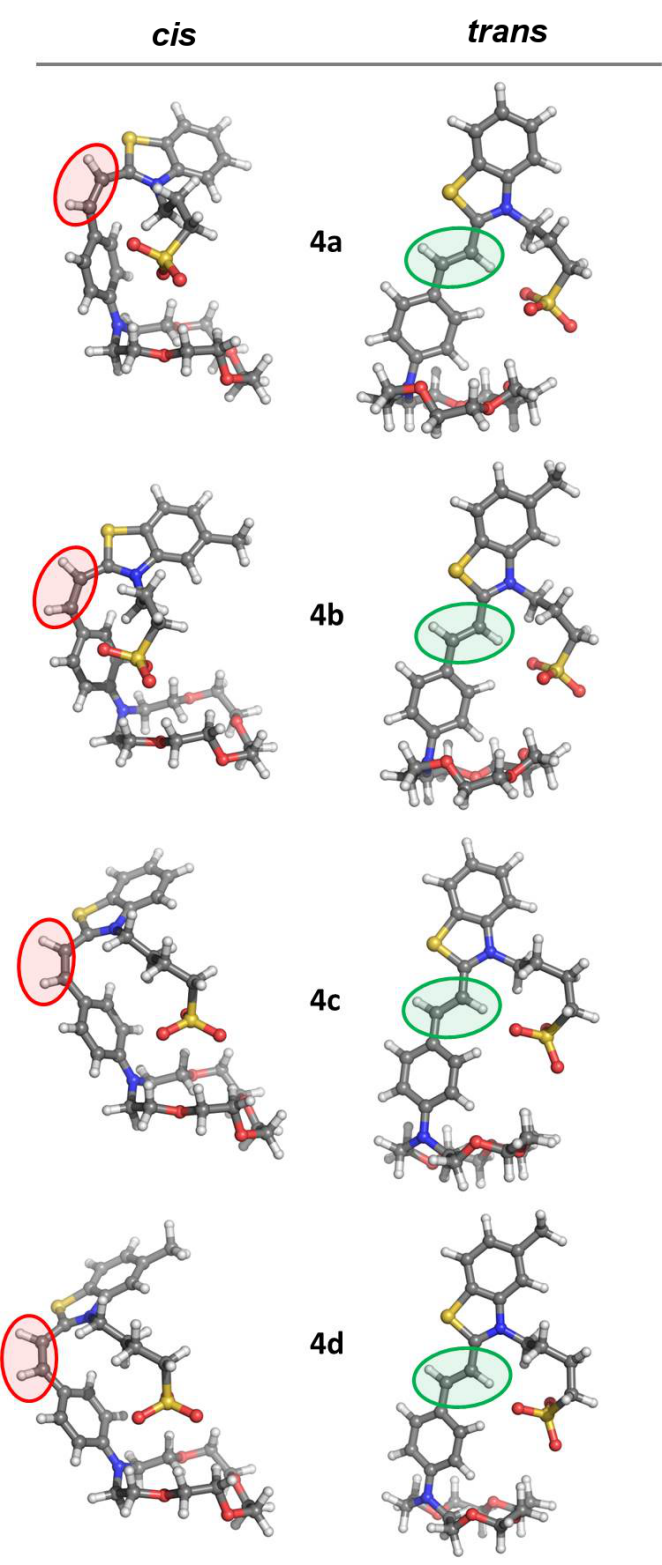
Optimized structures of the *cis* and *trans* isomers of dyes **4a–d**.

To rationalize the experimental findings, we performed density functional theory (DFT) calculations at the B3LYP/6-31+G(d,p) level. The first step in the molecular modelling investigation was the optimization of the molecular structures of the *cis* and *trans* isomers of dyes **4a–d** (with the –(CH_2_)*_n_*SO_3_^−^ (*n* = 3, 4) tails oriented to be in proximity to the aza-15-crown-5 fragments) in the gas phase (Figure 5). The thermochemical data for these are calculated at 298.15 K and a pressure of 1 atm. In Table 3 we report the enthalpies (*H*) for the *cis* and *trans* isomers and the energy storage capacity (Δ*H*) calculated as the difference in enthalpy between the *cis and trans* isomers. B3LYP calculations in the gas phase predict the *trans* forms of the dyes **4a–d** to be more stable. The *cis* forms are higher in energy by only 3.3 and 3.0 kJ mol^−1^ for **4a** and **4b**, respectively. The enthalpy differences calculated for dyes **4c** and **4d** are much higher (69.3 kJ mol^−1^ (**4c**) and 67.9 kJ mol^−1^ (**4d**), respectively).

**Table 3:** Calculated enthalpies (*H*) for the *cis* and *trans* isomers and storage energies (Δ*H*) for the systems studied in the gas phase and in acetonitrile solution.

Compound	Δ*H*, kJ mol^−1^	
	
	gas phase	acetonitrile

**4a**	3.3	39.7
**4b**	3.0	42.9
**4c**	69.3	32.3
**4d**	67.9	31.8

**Figure 6:**
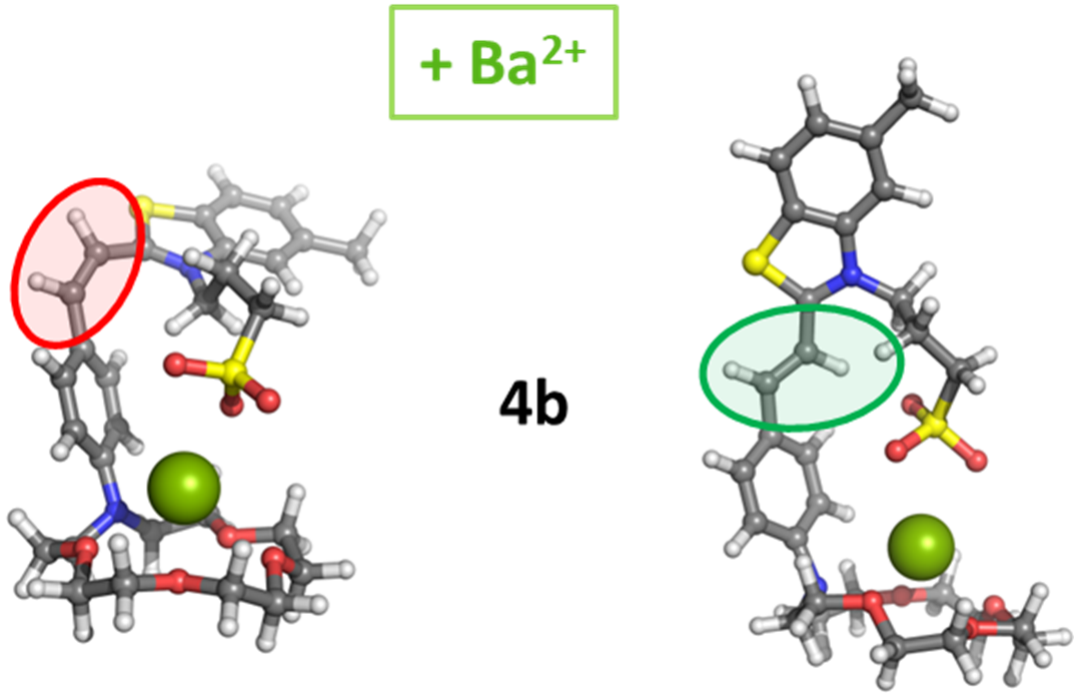
Optimized structures of the Ba^2+^-complexes of *cis* and *trans* isomer of **4b**.

Compounds **4a–d** can bind metal species at both isomeric forms. To determine the geometries of the 1:1 complexes with Ba^2+^ cations the metal cations were placed in the crown ether’s cavity and allowed to relax. In the optimized dye–Ba^2+^ complexes the metal cations are displaced “above” the crown ether’s cavity. Figure 6 depicts the gas-phase geometries of the Ba^2+^ complexes of the *cis* and *trans* isomer of **4b**.

The calculated enthalpies for the complex-formation reaction dye + Ba^2+^ → [dye–Ba]^2+^ in the gas phase, where dye = **4a**–**d**, with bare metal cations are listed in Table 4. The results obtained demonstrate that all reactions in the gas phase are predicted to be favorable. The ∆∆*H*^1^ values calculated for Ba^2+^ complex formation with the *trans* isomers of **4a–d** are more negative than the values obtained for the respective *cis* isomers.

**Table 4:** Calculated enthalpies, ∆∆*H*^1^, for [dye–Ba]^2+^ complex formation in the gas phase (in kJ mol^−1^).

Complex	∆∆*H*^1^

[*cis*-**4a**–Ba]^2+^	−793.3
[*trans*-**4a**–Ba]^2+^	−838.4
[*cis*-**4b**–Ba]^2+^	−790.2
[*trans*-**4b**–Ba]^2+^	−843.2
[*cis*-**4c**–Ba]^2+^	−759.0
[*trans*-**4c**–Ba]^2+^	−805.3
[*cis*-**4d**–Ba]^2+^	−762.0
[*trans*-**4d**–Ba]^2+^	−785.2

The complex formation processes between dyes with shorter –(CH_2_)*_n_*SO_3_^−^ tail (*n* = 3) are characterized by more negative ∆∆*H*^1^ values than those calculated for the dyes equipped with longer tails (*n* = 4), a trend corresponding to the trends in the experimentally derived stability constants. Conventional solvent treatment by methods like the polarizable continuum model (PCM) did not provide a good quantitative agreement with the experimental stability constants [23] so the data for ∆∆*H* in acetonitrile are not provided.

Time-dependent density functional theory (TDDFT) calculations were used to probe the electronic reorganizations upon excitation. TDPBE0 calculations with the 6–31+G(d,p) basis set for all atoms (except for Ba) and with the Stuttgart-Dresden SDD effective core potential (ECP) basis set for Ba predict one intensive band for all compounds in the range of 400–500 nm. The calculated optical parameters such as the absorption maximum (λ_max_), oscillator strength (*f*) and frontier orbital energy levels for the *trans* isomers of **4a–d** are listed in Table 5. The positions and intensities of the bands are consistent with the experimentally observed ones. The first excited states are determined by HOMO (highest occupied molecular orbital) → LUMO (lowest unoccupied molecular orbital) transitions.

**Table 5:** TDDFT/PBE0 calculated absorption maxima (λ_max_), oscillator strength (*f*) HOMO and LUMO energies and energy difference (HOMO–LUMO gap, HLG) for the *trans* isomers of compounds **4a–d** in acetonitrile.

Compound	λ_max,_ nm	*f*	HOMO, eV	LUMO, eV	HLG, eV

**4a**	477	1.58	−5.71	−2.98	2.73
**4b**	481	1.63	−5.64	−2.93	2.71
**4c**	482	1.63	−5.58	−2.90	2.69
**4d**	483	1.65	−5.56	−2.87	2.69

**Figure 7:**
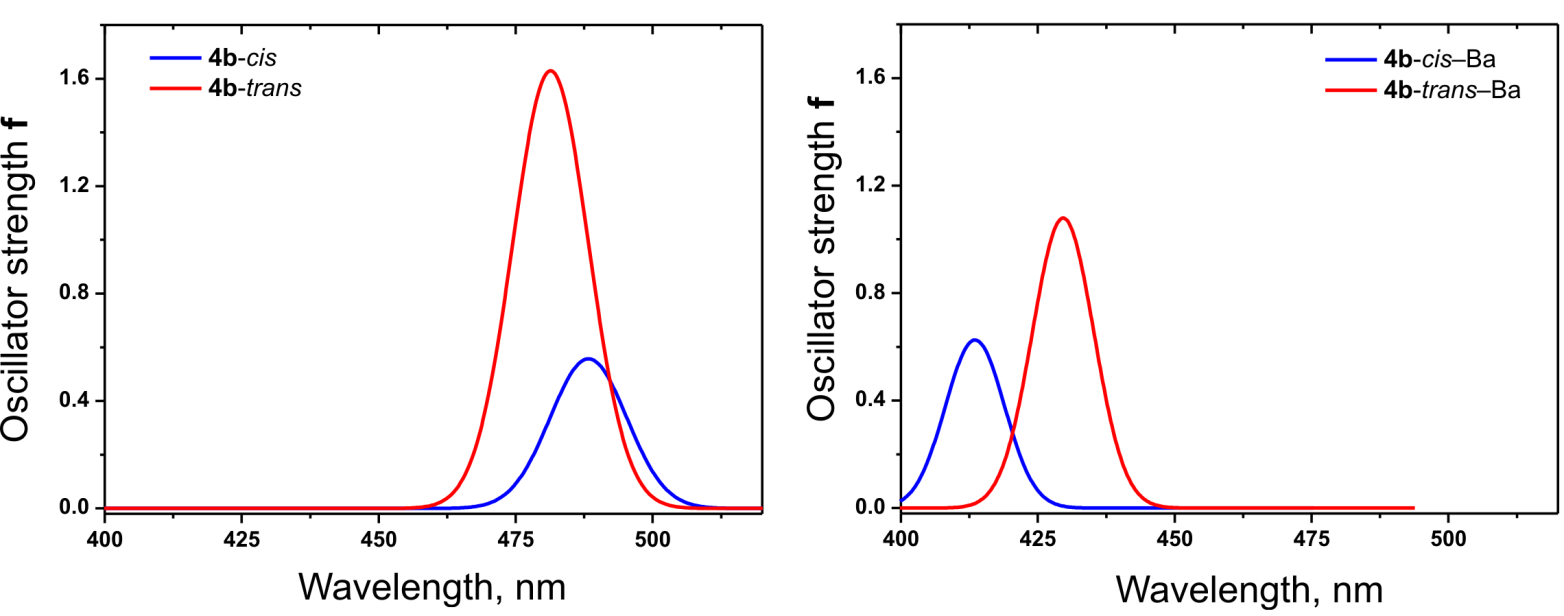
Simulated spectra with spectral lifetime broadening TDPBE0 spectra in ACN for dye **4b** and its Ba^2+^ complex.

The simulated spectra with spectral lifetime broadening (Gaussian function) with a full width at half-maximum (FWHM) of 0.15 eV and a height proportional to the oscillator strength for each transition spectrum for dye **4b** and its Ba^2+^ complex (Figure 7) are consistent with the experimental ones. The experimentally measured substantial hypo- and hypsochromic shift in the absorption spectra upon Ba^2+^ addition are also observed in the simulated spectra of the theoretically modeled structures of the dyes and the respective complexes.

The simulated spectra of the metal-free and Ba^2+^ complexed *cis* form of compound **4b** are also presented in Figure 7. The oscillator strengths of the *cis* forms, calculated at the same computational level, are found to be significantly lower than those calculated for the respective *trans* forms (metal-free compound *trans*-**4b** and *trans*-**4b**–Ba complex). These results correspond to the experimentally observed gradual increase in the intensity of the absorption maximum upon *cis-*to-*trans* reversion.

## Conclusion

Four benzothiazolium crown ether-containing styryl dyes (two known and two novel compounds) were synthesized through an optimized synthetic procedure. The photophysical properties of the new dyes were investigated in the absence and presence of Ba^2+^ cations and compared with those of the known dyes. The optimal conditions for the *trans*-to-*cis* photoisomerization of the styryl-crown ether containing dyes were identified. The dyes **4b** and **4d** with substituents in 5-position of the benzothiazole moiety demonstrated much better photophysical properties as molecular switches and MOST materials in comparison with the unsubstituted known analogs. The calculated thermodynamic changes associated with metal-ion complexation in the gas phase match the trends in the experimental stability constants.

## Experimental

### General

All solvents used in the present work were commercially available (HPLC grade). The starting materials **1a**, **1b**, **2a**, and **2b** were commercially available and were used as supplied. Melting points were determined on a Kofler apparatus and are uncorrected. NMR spectra were obtained on a Bruker Avance III 500 DRX 600 MHz spectrometer in DMSO-*d*_6_. The MALDI–TOF/TOF spectra were measured at Bruker “RapifeX” at MPIP, Germany. The stepwise experimental procedures for the synthesis of compounds **2–4** and characterization data are given in Supporting Information File 1. 4-(Aza-15-crown-5)benzocarbaldehyde (**3**) was synthesized using a slightly modified procedure [16–17].

UV–vis spectra were measured on a Unicam 530 UV–vis spectrophotometer in conventional quartz cells of 1 cm path length. The spectral bandwidth and the scan rate were 1 nm and 140 nm min^−1^, respectively. Stock solutions of each compound were prepared in spectroscopic grade acetonitrile (ACN) and all experiments were carried out in red light and at room temperature. Complex formation of dyes with Ba(ClO_4_)_2_ was studied by spectrophotometric titration. In the experiment aliquots of a solution containing known concentrations of dyes and of Ba(ClO_4_)_2_ were added to a solution of dyes alone at the same concentration. So the absorption spectra were recorded for solutions with identical total dye concentration (1 × 10^−5^ M) and variable total Ba(ClO_4_)_2_ concentration ranging from 1 × 10^−5^ M to 5 × 10^−1^ M in ACN.

Emission spectra were recorded on FluoroLog3-22, Horiba Jobin Yvon spectrofluorometer with Quanta–φ accessory having a large 150 mm integrating sphere for the quantum yield measurements. All spectra we recorded using quartz cells of 1 cm path length. The solution concentrations were chosen to give an absorbance ≤0.05 at the excitation wavelength of 440 and 488 nm.

The photoisomerization of the dyes and their complexes was performed by irradiating the samples in quartz cells (1 cm) with 16 mW laser (Qioptiq iFLEX2000-P-2-488) at λ = 488 nm for 3 min, a time which was found to be long enough to reach a photostationary state. The kinetics of the *cis*–*trans* thermal isomerization were studied by measuring the absorbance at a fixed wavelength in the dark as a function of time after irradiation stopped until the initial absorbance value before excitation was reached. The absorbance was measured by Ocean Optic HR2000+ high resolution USB fiber optic spectrometer fitted with 500 nm interference filter in the incident beam (10 nm bandwidth). During the irradiation and kinetic studies, the solutions were intensively magnetically stirred. A cuvette holder for fluorescent measurements was used allowing recording of the absorbance spectra during the laser irradiation in the perpendicular direction and immediately after the stop of irradiation.

### Computational details

Equilibrium geometries and intermolecular interaction energies for the host–guest assemblies between the dyes and metal cations were obtained by density functional theory (DFT) calculations using the B3LYP functional [25–26] (the most often used functional for organic molecules and complexes) and the 6-31+G(d,p) [27–29] basis set for the lighter atoms (C, O, S, N, H) and SDD pseudopotential for Ba atoms as implemented in the Gaussian 09 program package [30]. Frequency calculations for each optimized structure were performed at the same level of theory. No imaginary frequency was found for the lowest energy configurations of any of the optimized structures. In order to take into account the solvent effect induced by the acetonitrile solvent environment, the equilibrium geometries of the host–guest constituents and complexes were reoptimized considering the PCM (polarizable continuum model) solvent model [31]. The so-called basis set superposition error (BSSE) was not taken into account for the geometry optimization and intermolecular energy calculation. Time-dependent density functional theory calculations (TDDFT) using Perdew–Burke–Ernzerhof exchange-correlation functional (PBE0) were performed to compute the 10 lowest excited states of each structure [6–31+G(d,p) basis set for all atoms except Ba]. PyMOL molecular graphics system was used for generation of the molecular graphics images [32].

## Supporting Information

File 1 Experimental procedures for the synthesis of compounds **2–4** and characterization data of the new compounds.
