# Supplementary material for: Precious metal-free molecular machines for solar thermal energy storage
Source: Beilstein J Org Chem. 2019 May 14;15:1096–106. doi: 10.3762/bjoc.15.106 (PMC6541326; doi:10.3762/bjoc.15.106)
Supplement: File 1 — Experimental procedures for the synthesis of compounds 2–4 and characterization data of the new compounds. [file Beilstein_J_Org_Chem-15-1096-s001.pdf]

BEILSTEIN JOURNAL OF ORGANIC CHEMISTRY

Supporting Information

for

Precious metal-free molecular machines for solar thermal energy storage

Meglena I. Kandinska, Snejana M. Kitova, Vladimira S. Videva, Stanimir S. Stoyanov, Stanislava B. Yordanova, Stanislav B. Baluschev, Silvia E. Angelova and Aleksey A. Vasilev

Beilstein J. Org. Chem. **2019,** *15,* 1096–1106. doi:10.3762/bjoc.15.106

Experimental procedures for the synthesis of compounds 2–4 and characterization data of the new compounds

License and Terms: This is a supporting information file under the terms of the Creative Commons Attribution License (http://creativecommons.org/licenses/by/4.0). Please note that the reuse, redistribution and reproduction in particular requires that the authors and source are credited.
The license is subject to the *Beilstein Journal of Organic Chemistry* terms and conditions: (https://www.beilstein-journals.org/bjoc)

Table of contents

Synthesis and characterization of compounds **2–4**

Figures S1–S10, copies of ^{1}H and ^{13}C DEPT135 NMR spectra

Synthesis of quaternary benzothiazolium salts 2a-2d

The appropriate 2-methylbenzothiazole **1a** or **1b** (0.01 mol) and 0.01 mol 1,3-propansultone (**1c**) or 0.01 mol 1,4-butansultone (**1d**) were heated in a sealed tube under argon at 145 °C for 2 h. After cooling to room temperature 5 mL methanol were added and the solution was heated to reflux for 10 min. After cooling to room temperature the solution was poured into a mixture of 20 mL acetone and 20 mL diethyl ether. The formed precipitate was filtered, washed with two portions of 20 mL acetone and two portions of 20 mL diethyl ether and the precipitates were stored in a desiccator.

3-(2-Methylbenzo[*d*]thiazol-3-ium-3-yl)propane-1-sulfonate (2a): Yield 81%. ^{1}H-NMR (500 MHz, DMSO-$_{d6}$, δ(ppm)): 2.15-2.18 m (2H, CH_2), 2.64 t (2H, J^{3}_{HH} = 6.5 Hz, $CH_2SO_3^-$), 3.21 s (3H, CH_3), 4.93 t (2H, J^{3}_{HH} = 8.1 Hz,CH_2N^+), 7.78-7.82 m (1H, Ar), 7.88-7.91 m (1H, Ar), 8.43 d (1H, J^{3}_{HH} = 8.9 Hz, Ar), 8.45 d (1H, J^{3}_{HH} = 8.8 Hz, Ar). **3-(2,5-Dimethylbenzo[*d*]thiazol-3-ium-3-yl)propane-1-sulfonate (2b):** Yield: 93%. ^{1}H-NMR (500 MHz, DMSO-$_{d6}$, δ(ppm)): 2.09-2.16 m (2H, CH_2), 2.5 t (2H, J^{3}_{HH} = 6.4 Hz, $CH_2SO_3^-$), 2.51 s (3H, CH_3), 2.65 s (3H, CH_3), 4.87 t (2H, J^{3}_{HH} = 8.0 Hz,CH_2N^+), 7.63 s (1H, Ar), 8.27-8.29 m (2H, Ar).

4-(2-Methylbenzo[*d*]thiazol-3-ium-3-yl)butane-1-sulfonate: Yield 90%. ^{1}H-NMR (500 MHz, DMSO-$_{d6}$, δ (ppm)): 1.76-1.82 m (2H, CH_2), 1.95-2.01 m (2H, CH_2), 2.53 t (2H, J^{3}_{HH} = 7.2 Hz, $CH_2SO_3^-$), 3.23 s (3H, CH_3), 4.75 t (2H, J^{3}_{HH} = 8.1 Hz,CH_2N^+), 7.78-7.82 m (1H, Ar), 7.87-7.90 m (1H, Ar), 8.41-8.43 m (2H, Ar).

4-(2,5-Dimethylbenzo[*d*]thiazol-3-ium-3-yl)butane-1-sulfonate (2d): Yield 91%. ^{1}H-NMR (500 MHz, DMSO-$_{d6}$, δ (ppm)): 1.75-1.77 m (2H, CH_2), 1.79-1.97 m (2H, CH_2), 2.58 t (2H, J^3_{HH} = 7.2 Hz, $CH_2SO_3^-$), 3.22 s (3H, CH_3), 3.28 s (3H, CH_3), 4.68 t (2H, J^3_{HH} = 8.0 Hz,CH_2N^+), 7.62 d (1H, J^3_{HH} = 6.5 Hz, Ar), 8.25-8.28 m (2H, Ar).

Synthesis of *N*,*N*-bis(acetoxyethyl)phenylamine (2f) [1,2]

A mixture of *N*-phenyldiethanolamine (8.0 g, 44 mmol), triethylamine (13.3 g, 132 mmol) and THF (40 mL) was added to a solution of acetyl chloride (9.3 mL, 132 mmol) in THF (40 mL). The mixture was stirred at 35 °C for 20 h. Then, 40 mL water were slowly added dropwise to the mixture. After removal of THF by rotary evaporation, the mixture was extracted with chloroform and the organic layer was dried ($MgSO_4$) and concentrated under reduced pressure. The oily residue was pure enough to be used in the next reaction step. Yield: 77%. ^{1}H-NMR (500 MHz, DMSO-$_{d6}$, δ (ppm)): 2.04 s (6H, CH_3), 3.61 t (4H, J^3_{HH} = 12.6 Hz, CH_2), 4.23 t (4H, J^3_{HH} = 12.5 Hz, CH_2), 6.73–6.77 m (3H, Ar), 7.21 t (2H, J^3_{HH} = 16 Hz, Ar).

Synthesis of 4-(bis(2-acetoxyethyl)amino)benzaldehyde (2g)

4-(Bis(2-acetoxyethyl)amino)benzaldehyde (**2g**) was prepared using the experimental procedure and quantities described in ref. [3]: Yield 13 g, 99%. ^{1}H-NMR (500 MHz, DMSO-d_6, δ (ppm)): 2.04 (s, 6H, CH_3), 3.73 t (4H, J^3_{HH} = 12.3 Hz, CH_2), 4.27 t (4H, J^3_{HH} = 12.2 Hz, CH_2), 7.75 d (2H, J^3_{HH} = Hz, Ar), 6.82 d (2H, J^3_{HH} = 8.6 Hz, Ar), 9.73 s (H, CHO).

Synthesis of 4-(bis(2-hydroxyethyl)amino)benzaldehyde (2h)

A mixture of **2g** (10 g, 34 mmol), methanol (150 mL) and sodium carbonate solution (10.9 g, 102 mmol) was stirred at room temperature overnight [1,2,4]. A few drops of 1 N HCl were added and methanol was removed by rotary evaporation. The mixture was extracted with chloroform and the organic layer was dried (MgSO4) and concentrated under reduced pressure. The residue was purified by column chromatography eluting with ethyl acetate to give compound **2h** in a yield of 6.3 g, (90%). ^{1}H NMR (500 MHz, $CDCl_3$, TMS, δ (ppm)): 3.67 t (4H, J^3_{HH} = 5.0 Hz, CH_2O), 3.88 t (4H, J^3_{HH} = 5.0 Hz, CH_2O), 6.69 d (2H, J^3_{HH} = 8.9 Hz, Ar), 7.64 d (2H, J^3_{HH} = 8.7 Hz, Ar), 9.61 s (1H, CHO).

Synthesis of 4-(aza-15-crown-5)benzocarbaldehyde (3) [1,2]

A mixture of compound **2h** (2 g, 10.45 mmol) and NaH (4 g, 60% in mineral oil, 0.1 mol) in dry THF (300 mL) was refluxed under a nitrogen atmosphere for 30 min. The mixture was added dropwise to a solution of compound **2i** (1.0 g, 2.1 mmol) in THF (200 mL) and then stirred at 70 °C for 2 d. After adding an aqueous solution of H_2SO_4 (2 M, 50 mL), THF was removed by rotary evaporation. The mixture was then extracted with chloroform and the organic layer was dried (MgSO4) and concentrated under reduced pressure. The crude product was purified by column chromatography (eluent: acetone/*n*-hexane 3:2) to give **3** in a yield of 1.5 g, (45%). ^{1}H NMR (500 MHz, $CDCl_3$, TMS, δ (ppm)): 3.52-3.72 m (16H, CH_2O), 3.78-3.80 t (4H, NCH_2), 6.71 d (2H, J^3_{HH} = 8.7 Hz, Ar), 7.70 d (2H, J^3_{HH} = 8.5 Hz, Ar), 9.76 s (1H, CHO).

Synthesis of dyes 4a–d

General procedure

Intermediates **2a–d** (0.001 mol) and 0.0011 mol of aldehyde **3** were dissolved in 15 mL ethanol and two drops of piperidine were added. The reaction mixture was refluxed for 2 h, cooled to room temperature and 20 mL of ethyl acetate were added. The formed precipitate was suction filtered and dried in a desiccator. The dyes were purified by precipitation from ethanol/ethyl acetate 1:3.

Synthesis of dye 4a

2-Methyl-3-(3-sulfopropyl)-benzo[*d*]thiazole (**2a**, 0.27 g, 0.001 mol) and 4-(monoaza-15-crown-5)benzaldehyde (**3**, 0.36 g, 0.0011 mol) and 2 drops of piperidine were mixed and elaborated as described in the General procedure. Yield 0.29 g (51%), ^{1}H NMR (500 MHz, $CDCl_3$, TMS, δ (ppm)): 2.15-2.19 m (2H, CH_2), 2.67 t (2H, J^3_{HH} = 6.1 Hz, $CH_2SO_3^-$), 3.52 brs (4H, CH_2), 3.56-3.59 m (8H, CH_2), 3.69-3.71 m (8H, CH_2), 4.97 t (2H, J^3_{HH} = 7.8 Hz, CH_2N^+), 6.83 d (2H, J^3_{HH} = 8.9 Hz, CH), 7.67 t (1H, J^3_{HH} = 7.8 Hz, CH), 7.77 t (1H, J^3_{HH} = 7.9 Hz, CH), 7.86 d (1H, J^3_{HH} = 15.2 Hz, CH=CH), 7.96 d (2H, J^3_{HH} = 8.6 Hz, CH), 8.10 d (1H, J^3_{HH} = 15.1 Hz, CH=CH), 8.23 d (1H, J^3_{HH} = 8.5 Hz, CH), 8.29 d (1H, J^3_{HH} = 8.0 Hz, CH). ^{13}C-NMR (*δ* (ppm), DMSO-d_6, DEPT 135 (125 MHz)): 25.26 (CH_2), 47.36 (CH_2), 47.70 (CH_2), 52.84 (CH_2O), 68.28 (CH_2O), 69.52 (CH_2O), 70.09 (CH_2O), 70.77 (CH_2O), 106.88 (CH), 112.52 (CH), 116.29 (CH), 124.38 (CH), 127.85 (CH), 129.42 (CH), 133.68 (CH), 150.94 (CH). IR (nujol) v_{max}: 1580, 1510, 1450, 1395, 1320, 1260, 1190, 1110, 1020, 810, 770, 510 cm-1; MS (MALDI-TOF): m/z (%): 577.13 (Elemental analysis for Mw = 576.72: Calc. C 58.31, H 6.29, N 4.86; Found C 57.81, H 5.94, N 4.79.

Synthesis of dye 4b

2,5-Dimethyl-3-(3-sulfopropyl)-benzo[*d*]thiazole (**2b**, 0.29 g, 0.001 mol) and 4-(monoaza-15-crown-5)benzaldehyde (**3**, 0.36 g, 0.0011 mol) and 2 drops of piperidine were mixed and elaborated as described in the General procedure. Yield 0.37 g (63%), ^{1}H NMR (500 MHz, $CDCl_3$, TMS, δ (ppm)): 2.15-2.18 m (2H, CH_2), 2.53 s (3H, CH_3), 2.66 t (2H, J^3_{HH} = 6.1 Hz, $CH_2SO_3^-$), 3.52 brs (4H, CH_2), 3.56-3.58 m (8H, CH_2), 3.68-3.71 m (8H, CH_2), 4.93 t (2H, J^3_{HH} = 7.4 Hz, CH_2N^+), 6.82 d (2H, J^3_{HH} = 8.9 Hz, CH), 7.50 d (1H, J^3_{HH} = 8.2 Hz, CH), 7.86 d (1H, J^3_{HH} = 15.2 Hz, CH=CH), 7.95 d (2H, J^3_{HH} = 8.7 Hz, CH), 8.04 d (1H, J^3_{HH} = 15.2 Hz, CH=CH), 8.09 s (1H, CH), 8.15 d (2H, J^3_{HH} = 8.3 Hz, CH). ^{13}C-NMR (*δ* (ppm), DMSO-d_6, DEPT 135 (125 MHz)): 21.66 (CH_3), 47.28 (CH_2), 47.70 (CH_2), 48.02 (CH_2), 52.83 (CH_2O), 68.28 (CH_2O), 69.52 (CH_2O), 70.09 (CH_2O), 70.77 (CH_2O), 107.03 (CH), 112.50 (CH), 116.21 (CH), 123.91 (CH), 129.20 (CH), 133.57 (CH), 133.64 (CH), 150.50 (CH), 177.52 (CH). IR (nujol) v_{max}: 1590, 1510, 1460, 1380, 1350, 1310, 1270, 1220, 1190, 1110, 1020, 820, 720, 580, 510 cm-1; MS (MALDI-TOF): m/z (%): 591.14. Elemental analysis for Mw = 590.75: Calc. C 58.96, H 6.48, N 4.74; Found C 58.72, H 6.15, N 4.71.

Synthesis of dye 4c

2-Methyl-3-(4-sulfobutyl)-benzo[*d*]thiazole (**2c**, 0.29 g, 0.001 mol), and 4-(monoaza-15-crown-5)benzaldehyde (**3**, 0.36 g, 0.0011 mol) and 2 drops of piperidine were mixed and elaborated as described in the General procedure. 0.32 g (Yield 55%), ^{1}H NMR (500 MHz, $CDCl_3$, TMS, δ (ppm)): 1.82-1.86 m (2H, CH_2), 1.93-1.98 m (2H, CH_2), 2.56 t (2H, J^3_{HH} = 7.1 Hz, $CH_2SO_3^-$), 3.52 brs (4H, CH_2), 3.56-3.59 m (8H, CH_2), 3.69-3.71 m (8H, CH_2), 4.82 t (2H, J^3_{HH} = 7.8 Hz, CH_2N^+), 6.84 d (2H, J^3_{HH} = 9.0 Hz, CH), 7.68 dd (1H, J^3_{HH} = 7.5 Hz, CH), 7.75 d (1H, J^3_{HH} = 15.2 H d (1H, J^3_{HH} = 15.2

Hz, CH=CH), 8.21 d (1H, J^3_{HH} = 8.5 Hz, CH), 8.29 d (1H, J^3_{HH} = 7.8 Hz, CH). IR (nujol) ν_{max}: 1580, 1510, 1450, 1360, 1350, 1320, 1310, 1280, 1220, 1190, 1120, 1110, 1020, 970, 920, 810, 720, 510 cm-1; MS (MALDI-TOF): m/z (%): 591.12. Elemental analysis for Mw = 590.75: Calc. C 58.96, H 6.48, N 4.74; Found C 58.99, H 6.31, N 4.68.

Synthesis of dye 4d

2,5-Dimethyl-3-(4-sulfobutyl)-benzo[*d*]thiazole (**2d**, 0.30 g, 0.001 mol) and 4-(monoaza-15-crown-5)benzaldehyde (**3**, 0.36 g, 0.0011 mol) and 2 drops of piperidine were mixed and elaborated as described in the General procedure. 0.45 g (Yield 74%), ^{1}H NMR (500 MHz, $CDCl_3$, TMS, δ (ppm)): 1.83-1.87 m (2H, CH_2), 1.94-1.98 m (2H, CH_2), 2.55 s (3H, CH_3), 3.01 t (2H, J^3_{HH} = 7.3 Hz, $CH_2SO_3^-$), 3.52 brs (4H, CH_2), 3.56-3.58 m (8H, CH_2), 3.69-3.72 m (8H, CH_2), 4.78 t (2H, J^3_{HH} = 7.6 Hz, CH_2N^+), 6.84 d (2H, J^3_{HH} = 8.9 Hz, CH), 7.52 d (1H, J^3_{HH} = 8.9 Hz, CH), 7.74 d (1H, J^3_{HH} = 15.1 Hz, CH=CH), 7.94 dd (2H, J^3_{HH} = 7.3 Hz, CH), 8.03-8.07 m (2H, CH=CH+CH_2), 8.16 d (1H, J^3_{HH} = 8.2 Hz, CH). IR (nujol) ν_{max}: 1580, 1510, 1460, 1400, 1380, 1320, 1320, 1290, 1280, 1190, 1180, 1110, 1010, 920, 810, 780 720, 590, 530 cm-1; MS (MALDI-TOF): m/z (%): 605.17. Elemental analysis for Mw = 604.78: Calc. C 59.58, H 6.67, N 4.63; Found C 58.72, H 6.15, N 4.71.

Figures S1–S10:

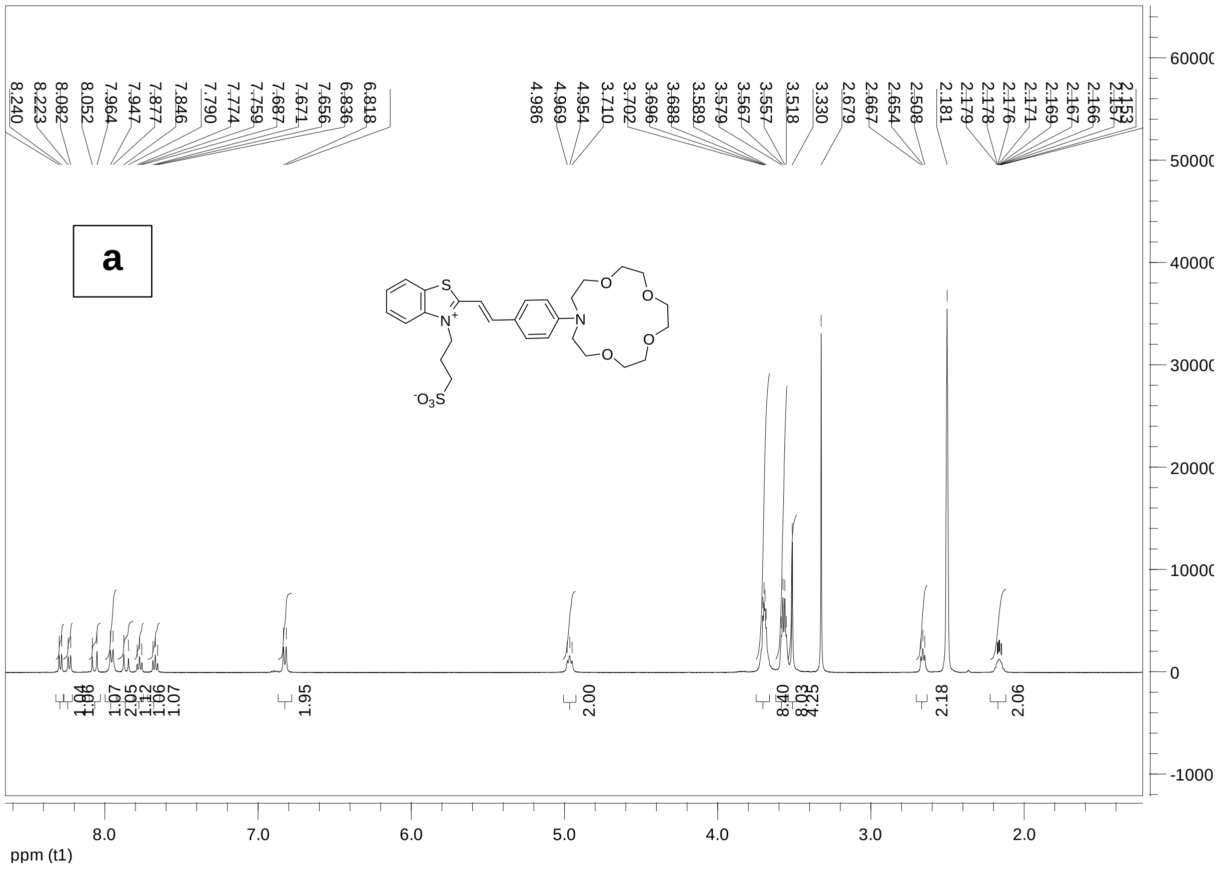

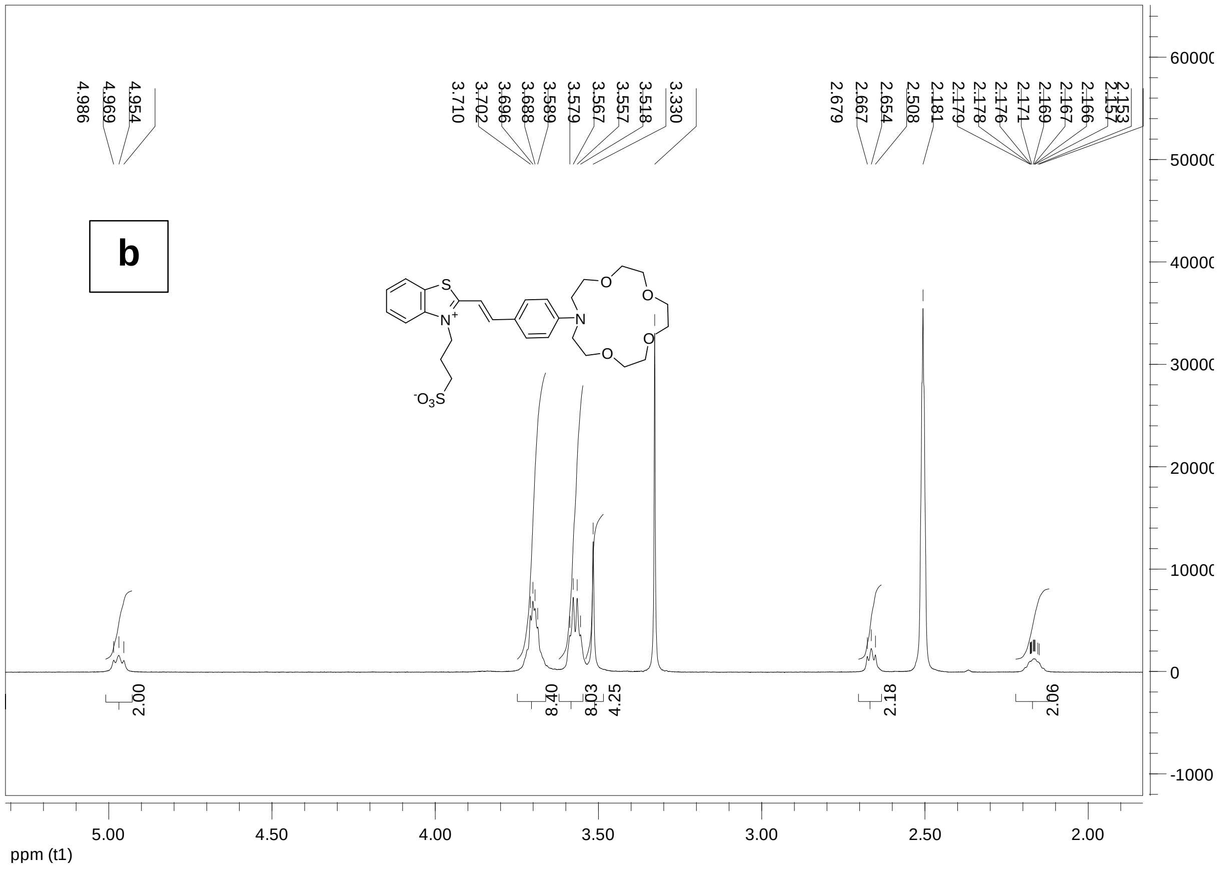

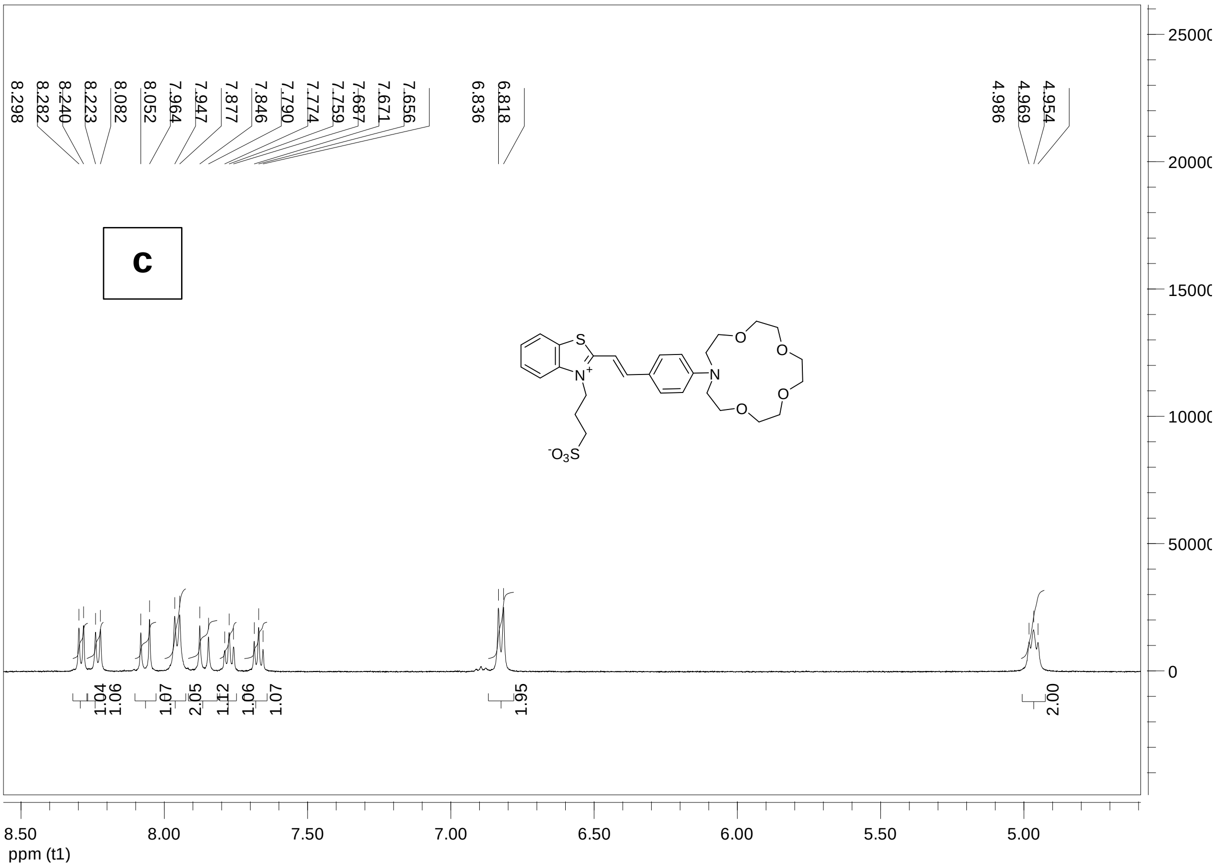

Figure S1 (a–c): ^{1}H NMR spectra of compound **4a**.

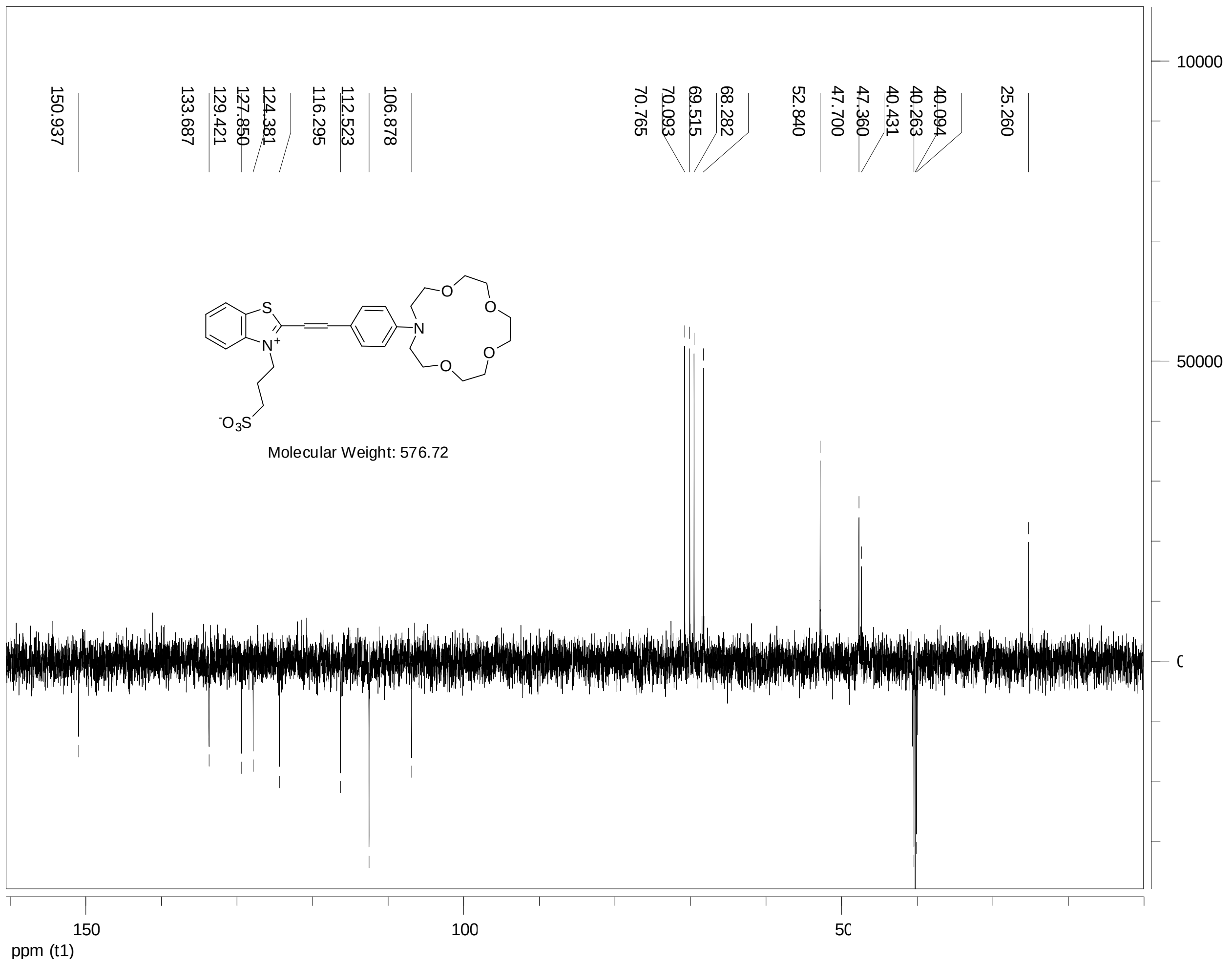

Figure S2: ^{13}C DEPT135 NMR spectrum of compound **4a**.

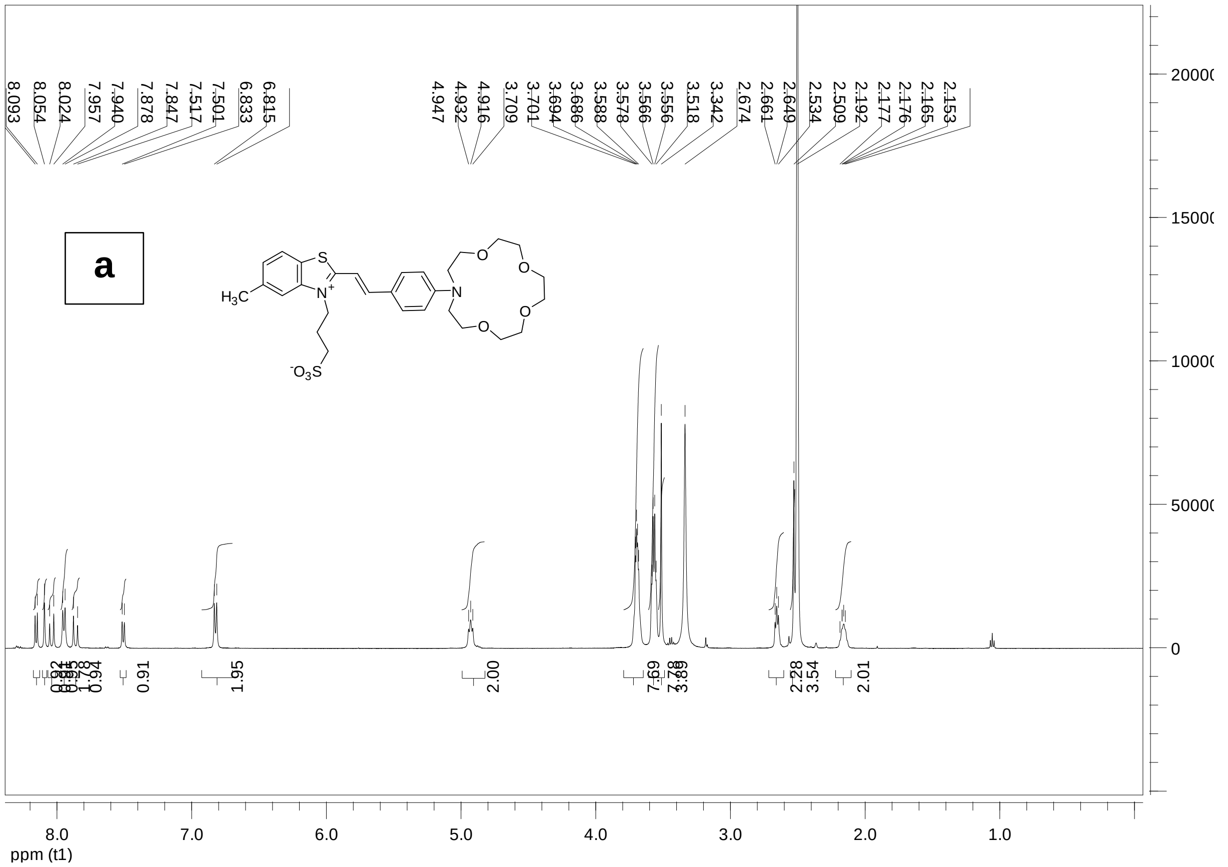
8.093 8.054 8.024 7.957 7.940 7.878 7.847 7.517 7.501 6.833 6.815
4.947 4.932 4.916 3.709 3.701 3.694 3.686 3.588 3.578 3.566 3.556 3.518 3.342 2.674 2.661 2.649 2.534 2.509 2.192 2.177 2.176 2.165 2.153
a
H_3C
S
N+
-O3S
N
O
0.91
1.95
2.00
7.69
7.78
3.89
2.28
3.54
2.01
20000
15000
10000
50000
0
8.0 7.0 6.0 5.0 4.0 3.0 2.0 1.0
ppm (t1)

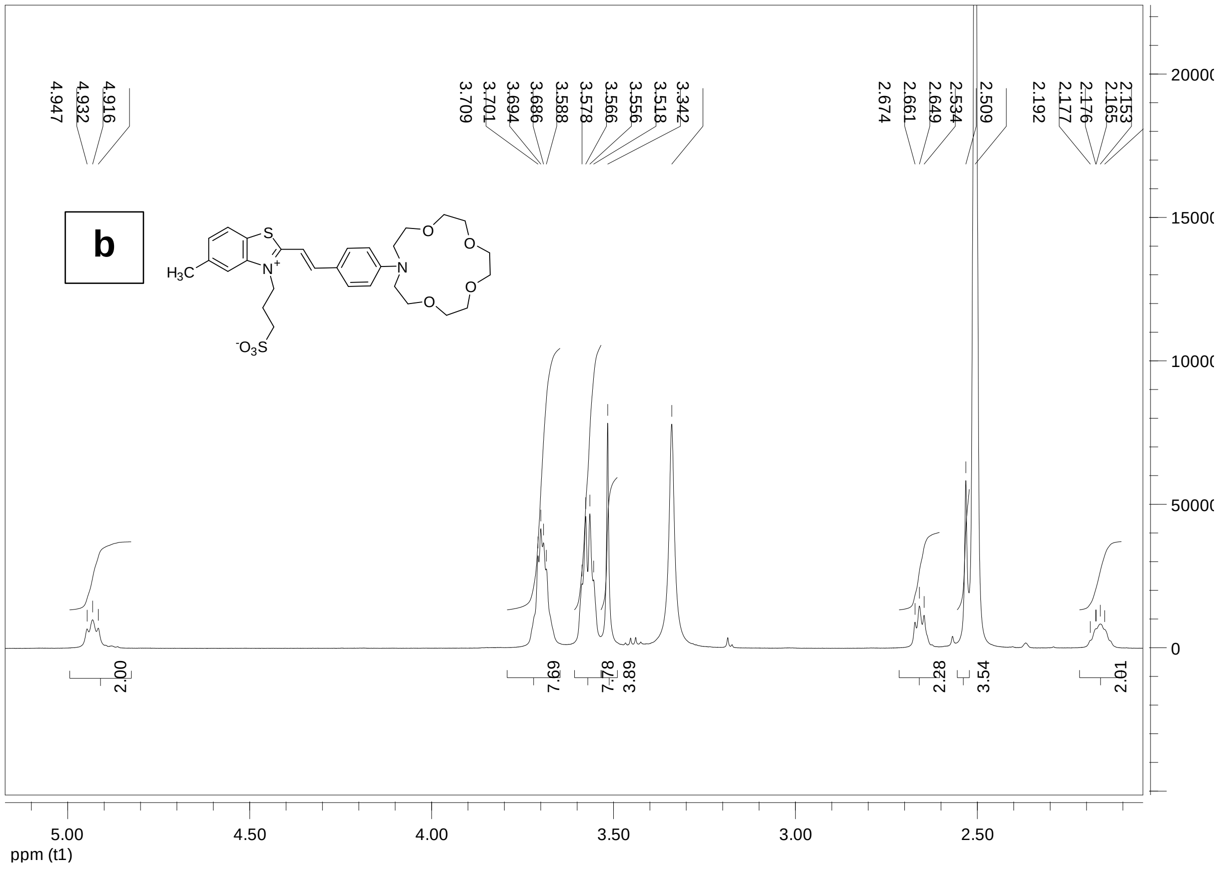
4.947 4.932 4.916
3.709 3.701 3.694 3.686 3.588 3.578 3.566 3.556 3.518 3.342
2.674 2.661 2.649 2.534 2.509 2.192 2.177 2.176 2.165 2.153
b
H_3C
S
N+
-O3S
N
O
2.00
7.69
7.78
3.89
2.28
3.54
2.01
20000
15000
10000
50000
0
5.00 4.50 4.00 3.50 3.00 2.50
ppm (t1)

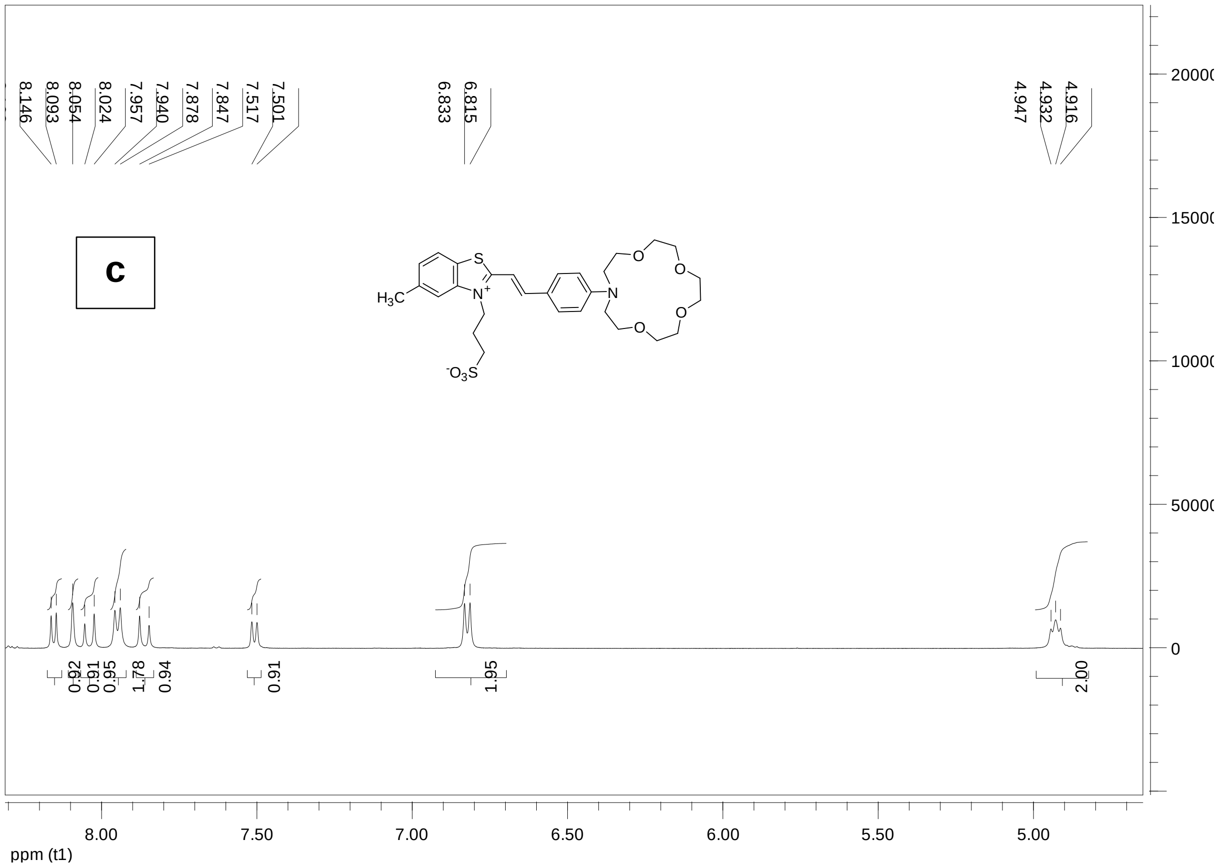

Figure S3 (a–c): ^{1}H NMR spectra of compound **4b**.

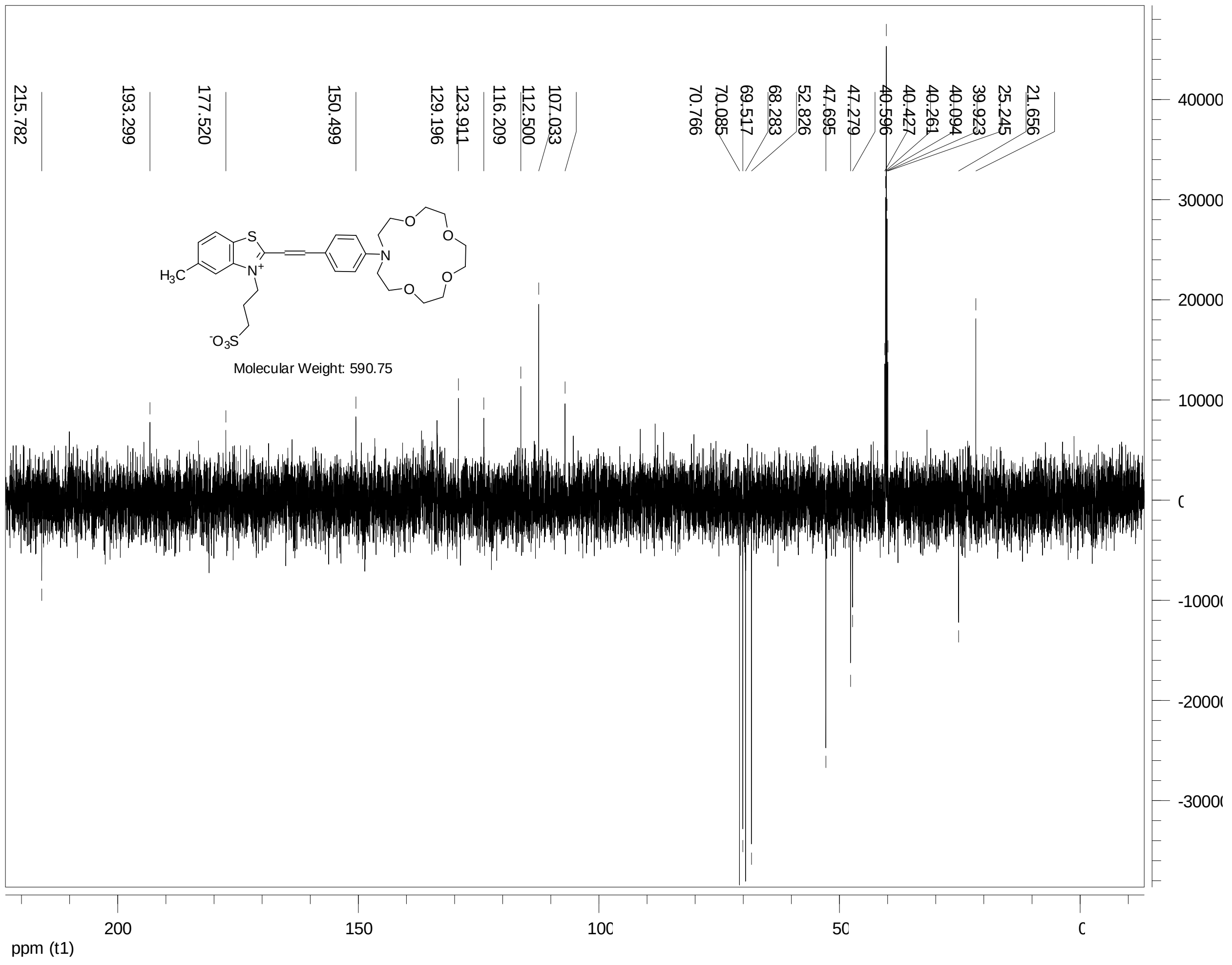

Figure S4: ^{13}C DEPT135 NMR spectrum of compound **4b**.

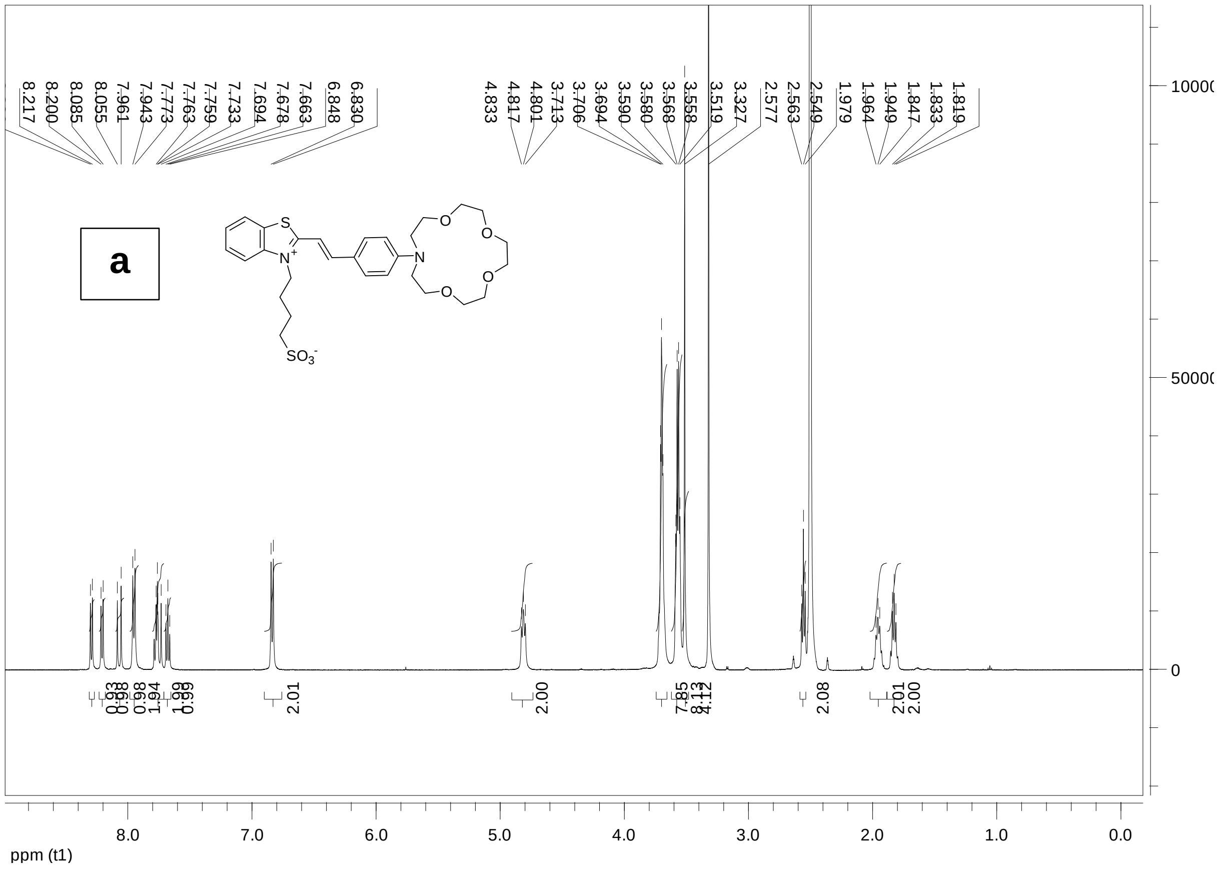

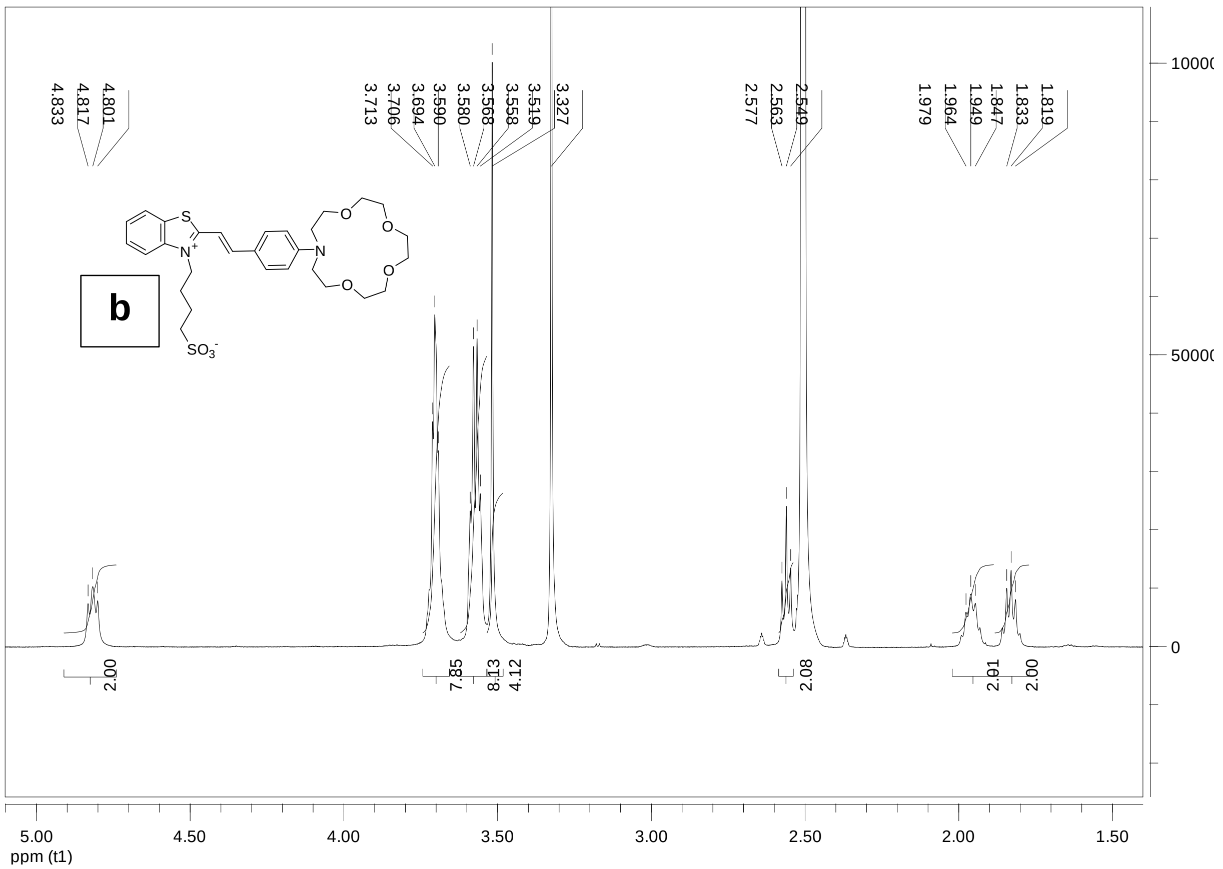

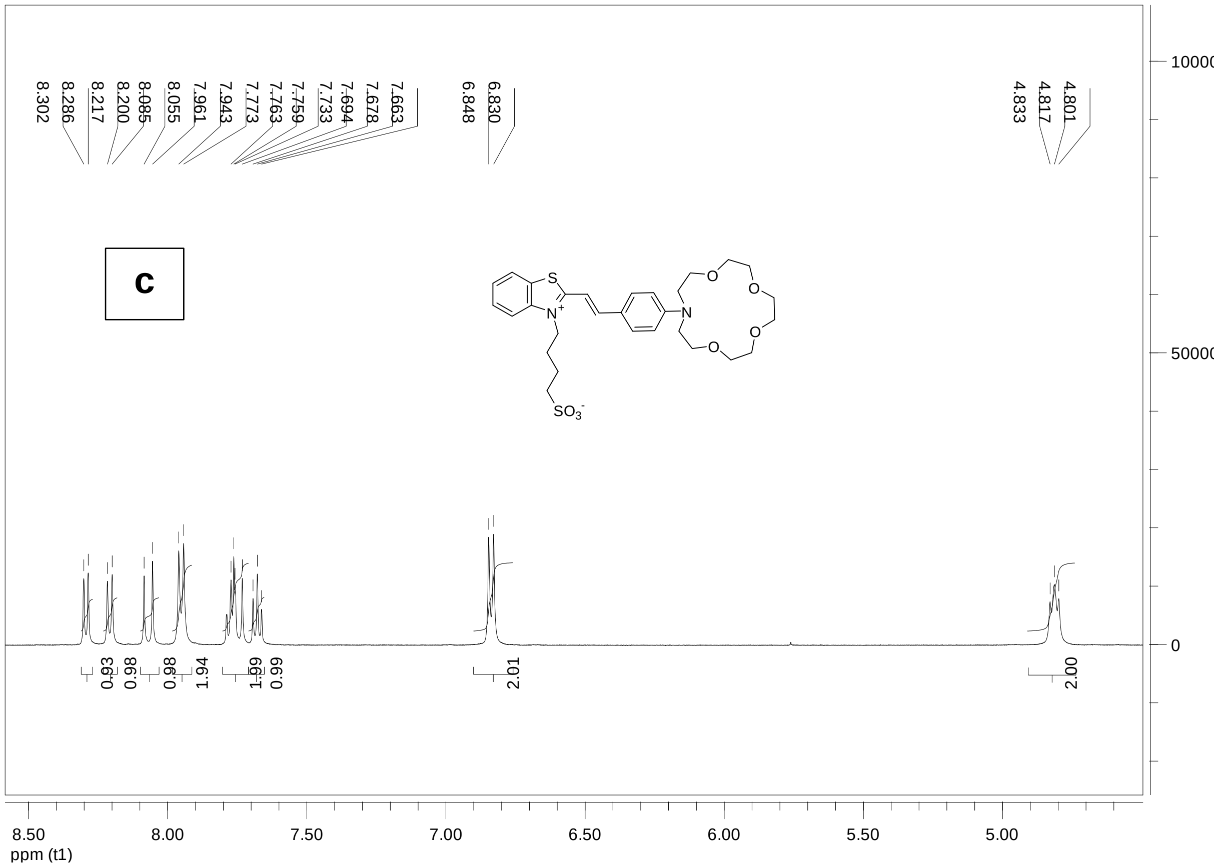

Figure S5 (a–c): ^{1}H NMR spectra of compound **4c**.

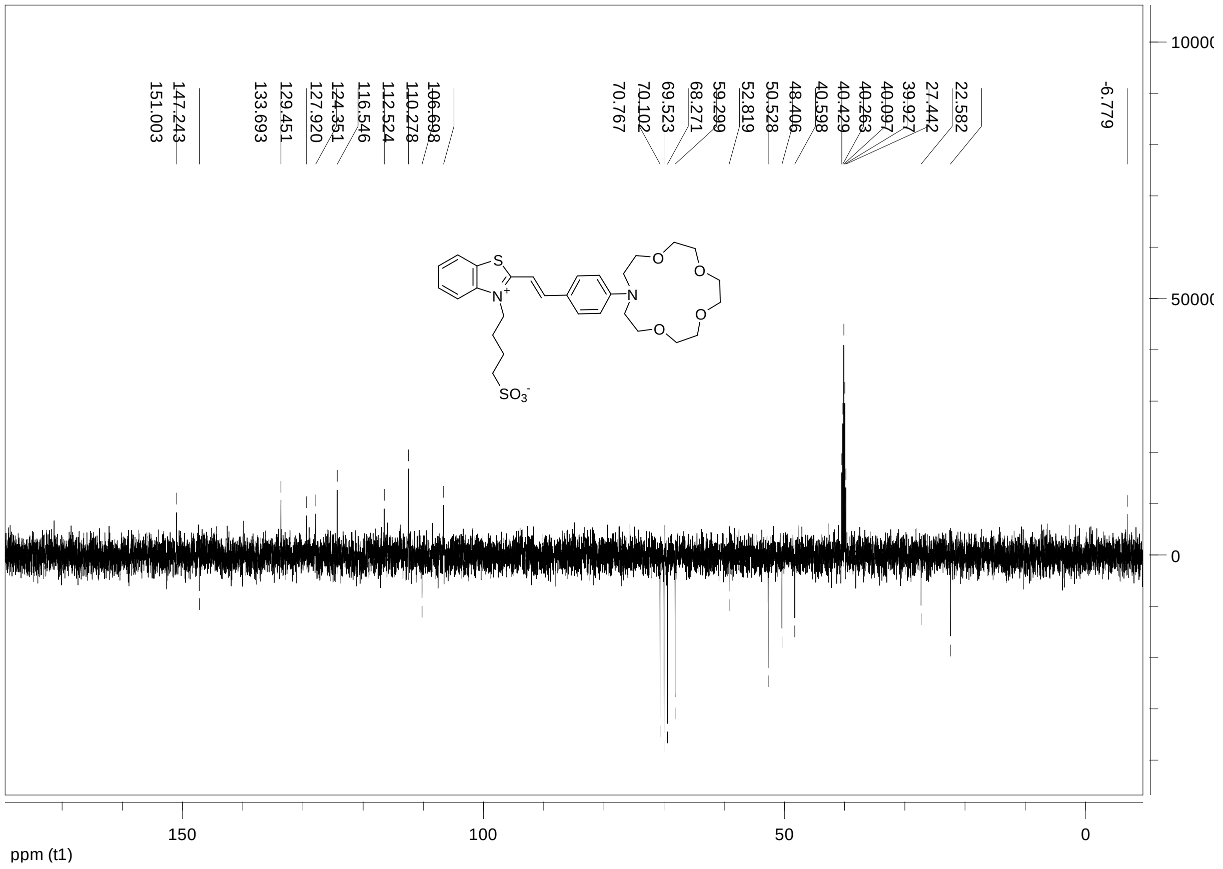

Figure S6: ^{13}C DEPT135 NMR spectrum of compound **4c**.

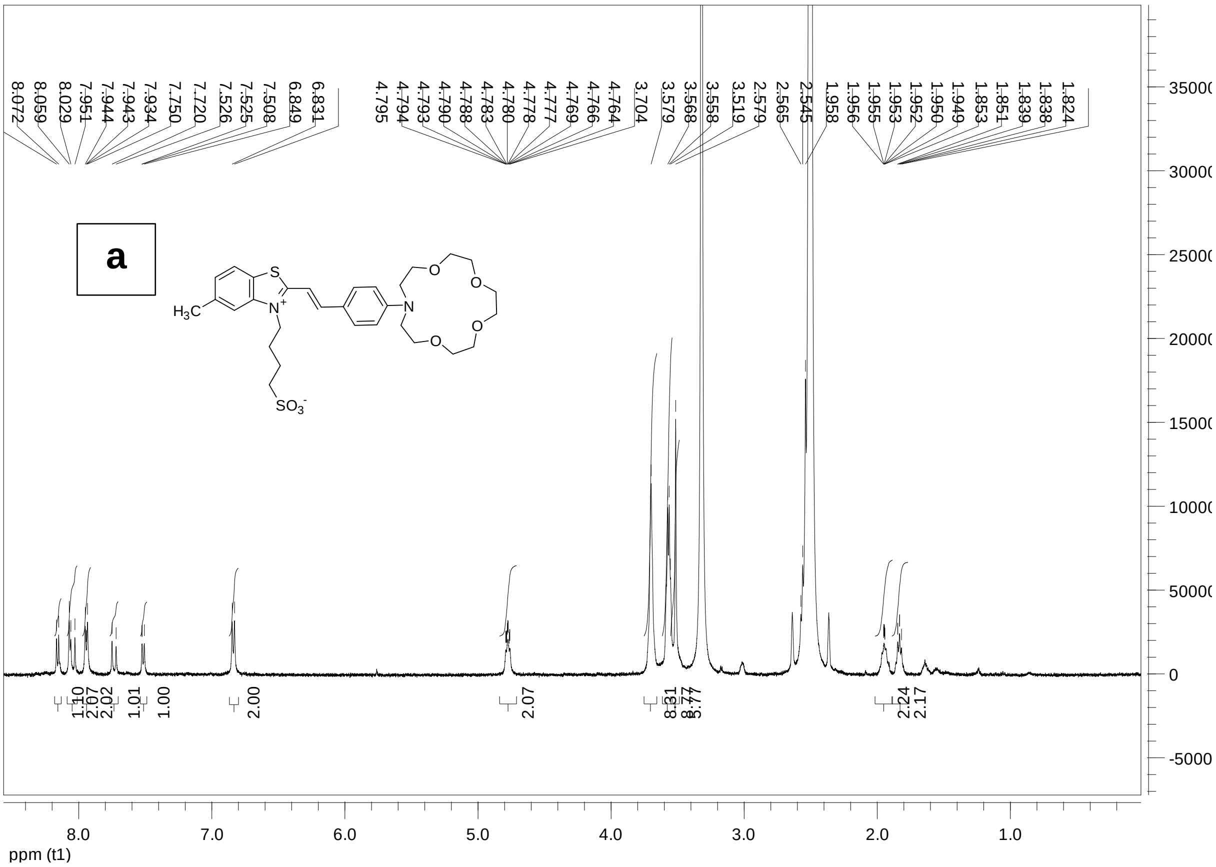
a
H_3C
S
N+
N
O
O
O
O
SO3-
8.0
7.0
6.0
5.0
4.0
3.0
2.0
1.0
ppm (t1)
1.10
2.07
2.02
1.01
1.00
2.00
2.07
8.31
8.77
5.77
2.24
2.17

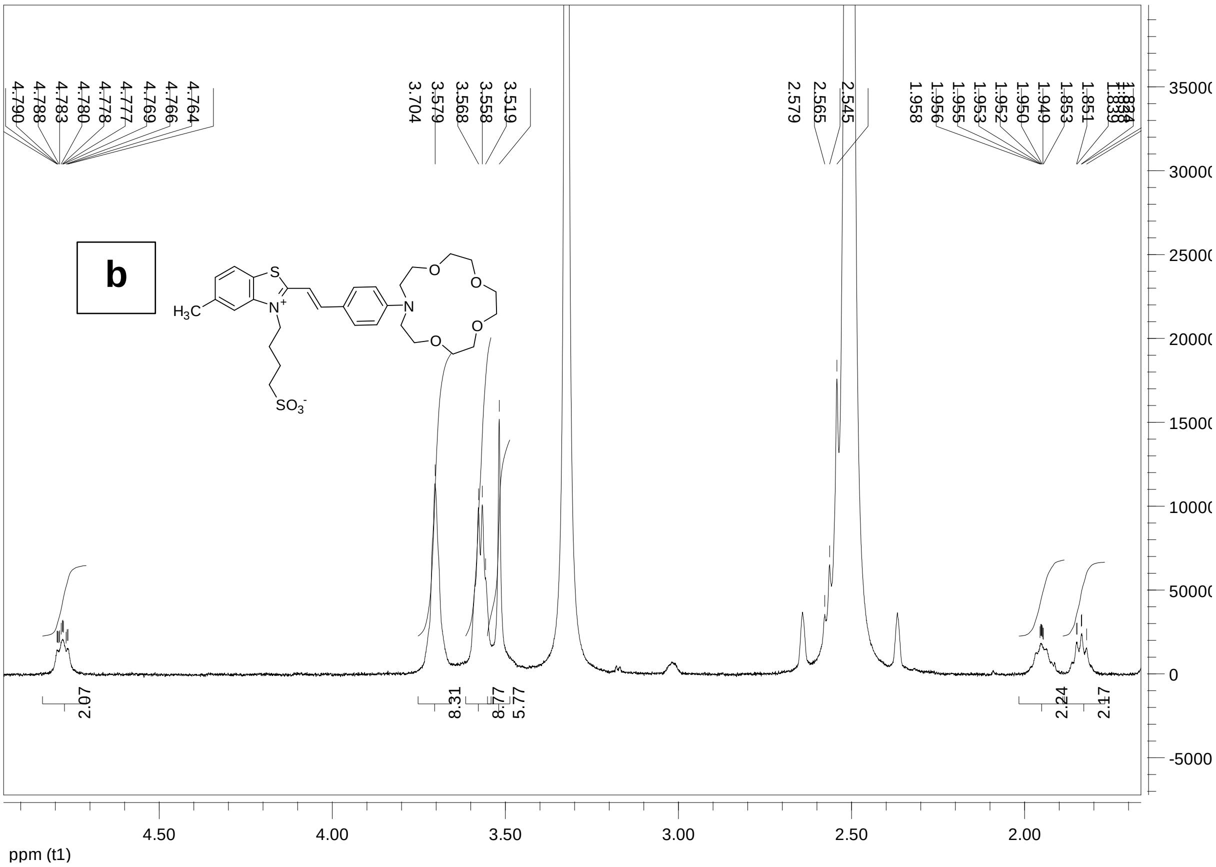
b
H_3C
S
N+
N
O
O
O
O
SO3-
4.50
4.00
3.50
3.00
2.50
2.00
ppm (t1)
2.07
8.31
8.77
5.77
2.24
2.17

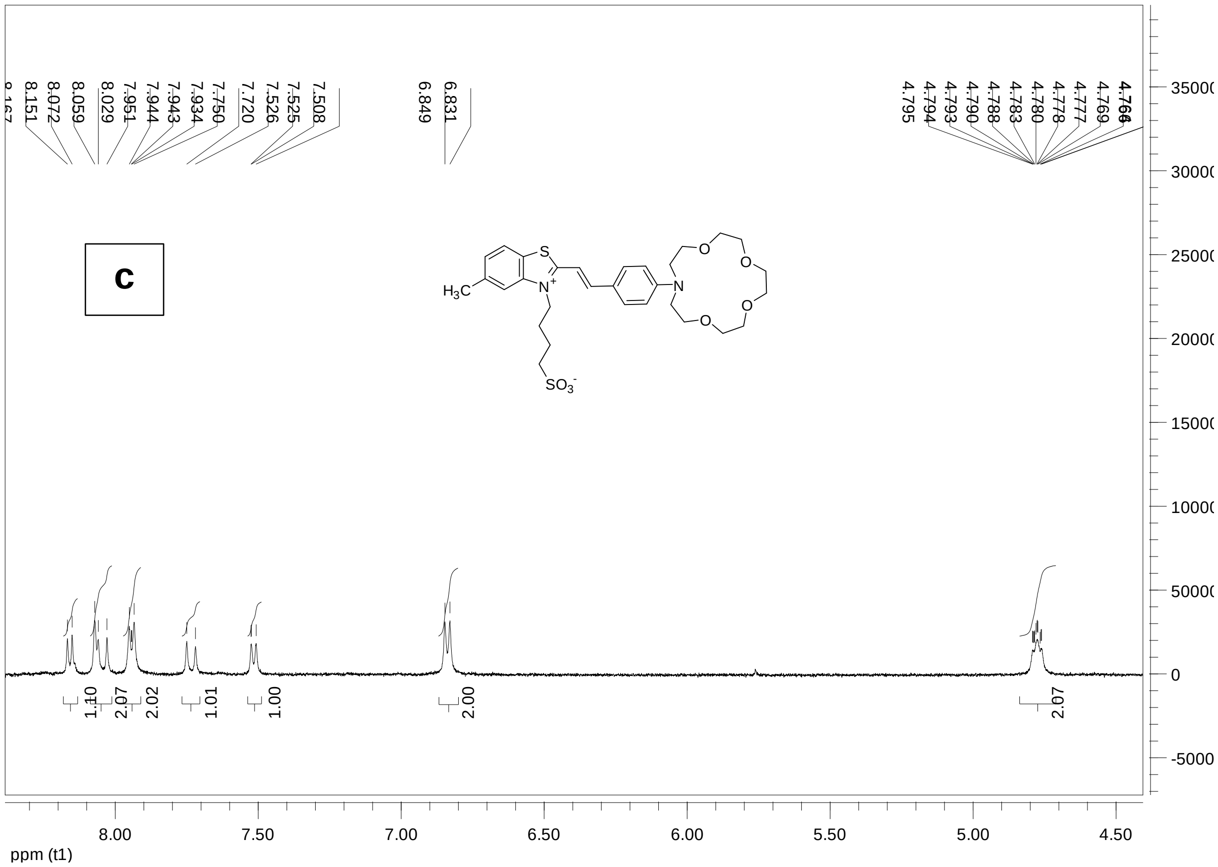

Figure S7 (a–c): ^{1}H NMR spectra of compound **4d**.

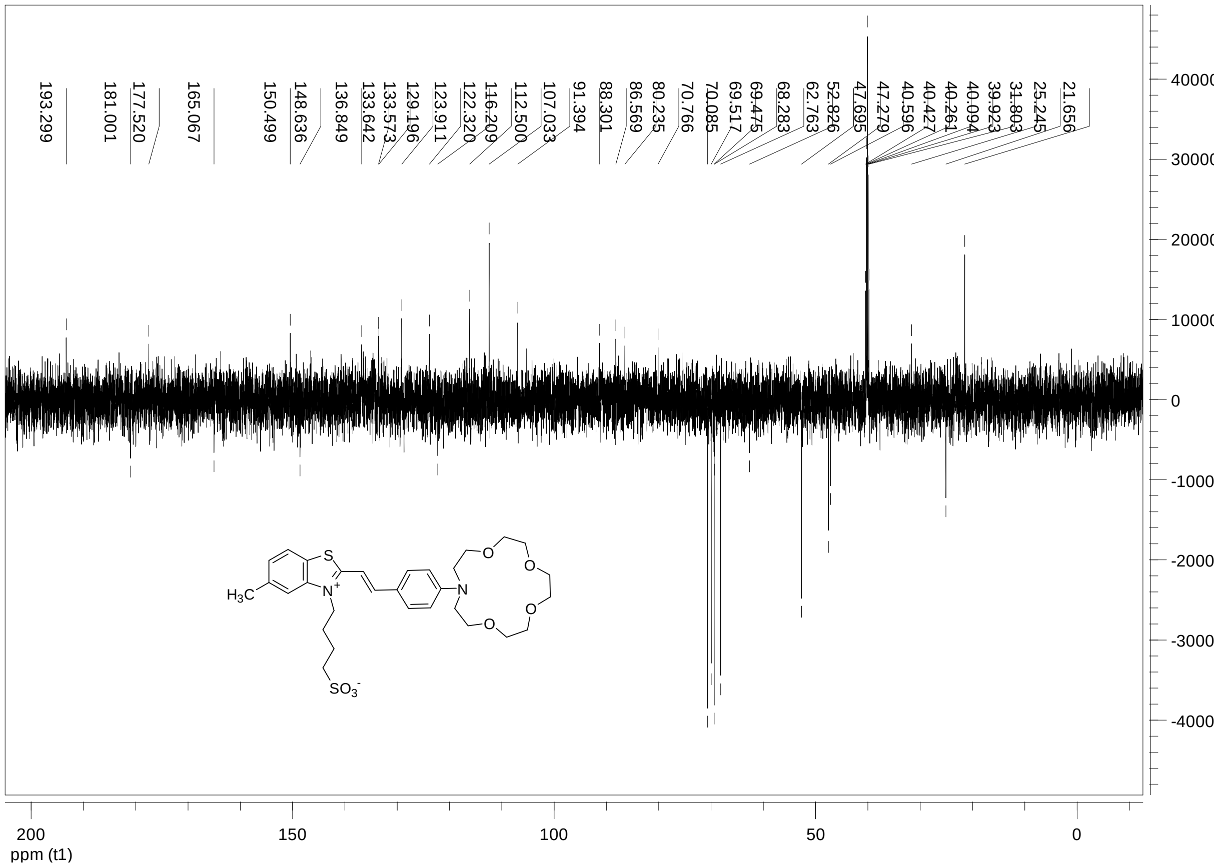

Figure S8: ^{13}C DEPT135 NMR spectrum of compound **4d** (very low solubility).

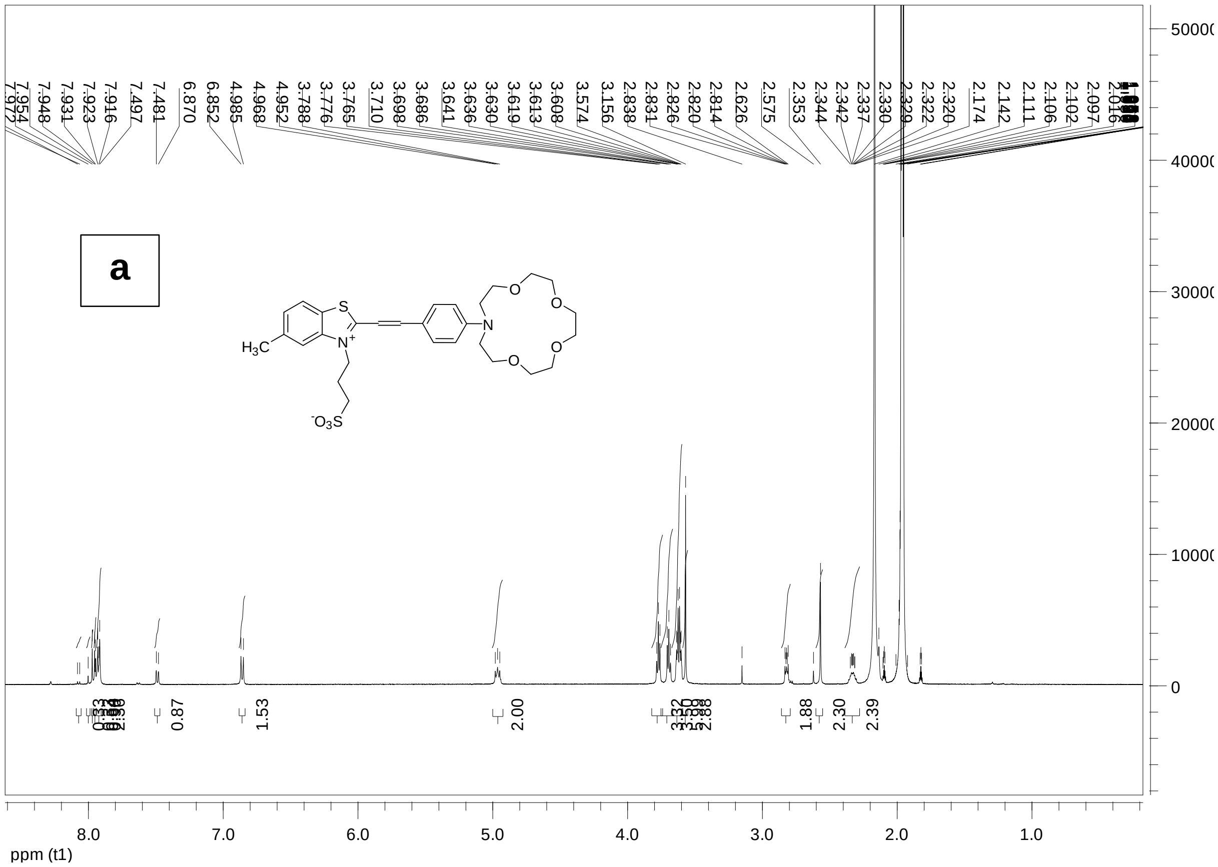

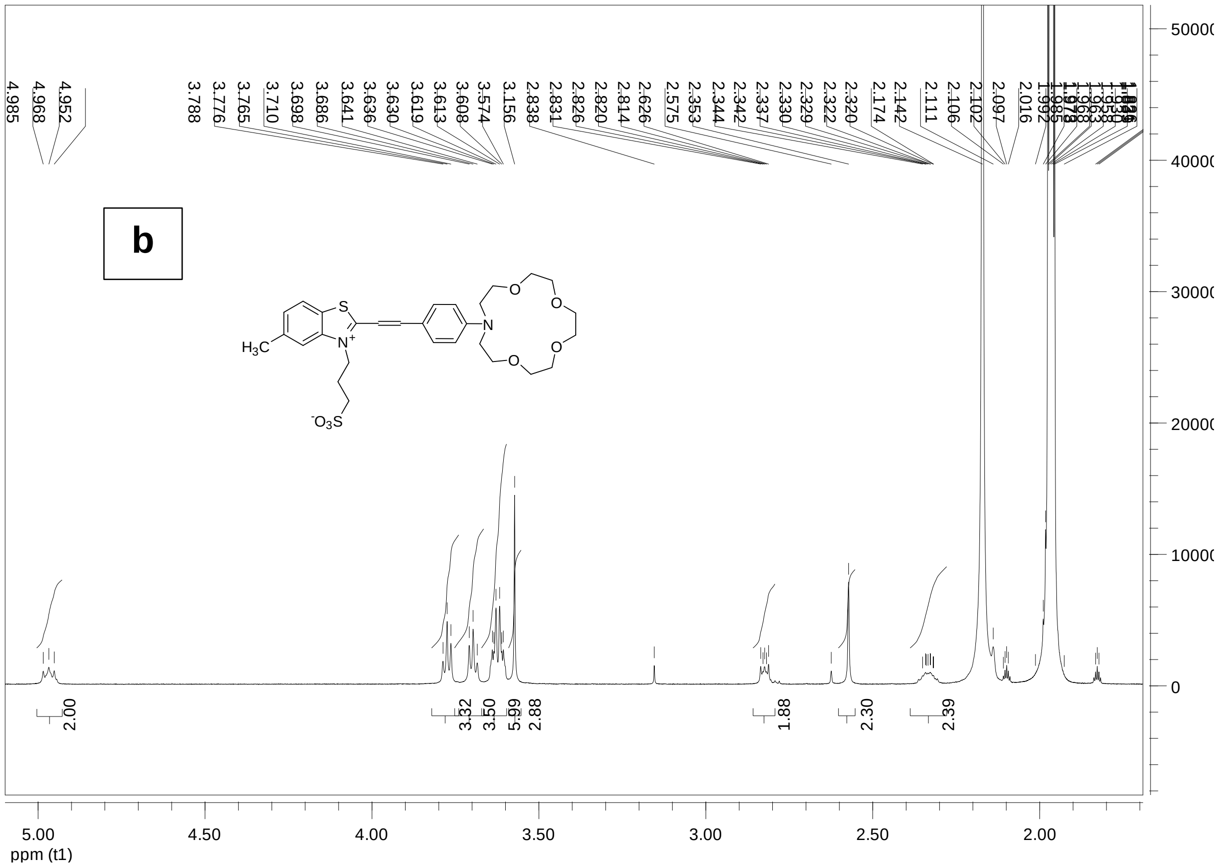

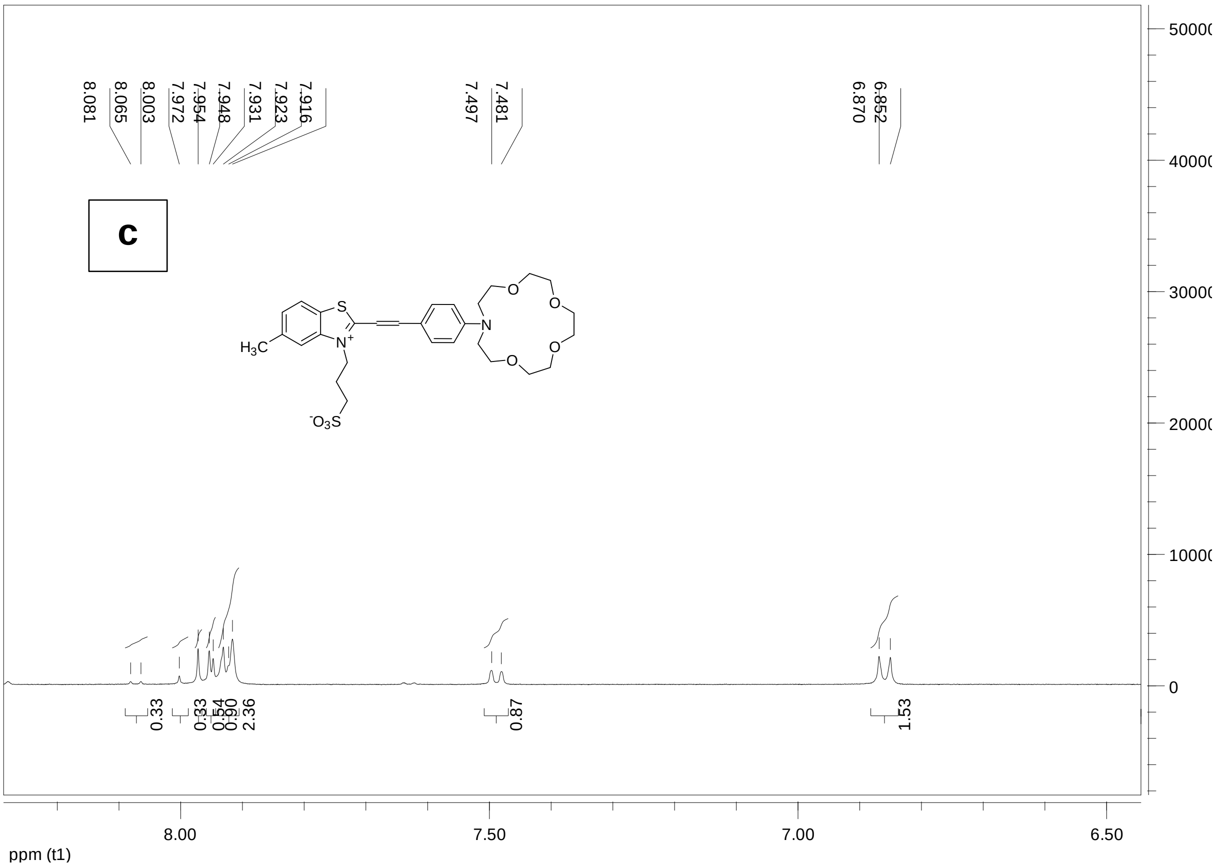

Figure S9 (a–c): ^{1}H NMR spectra of compound **4b (**1 × 10^{-3} M in CD_3CN).

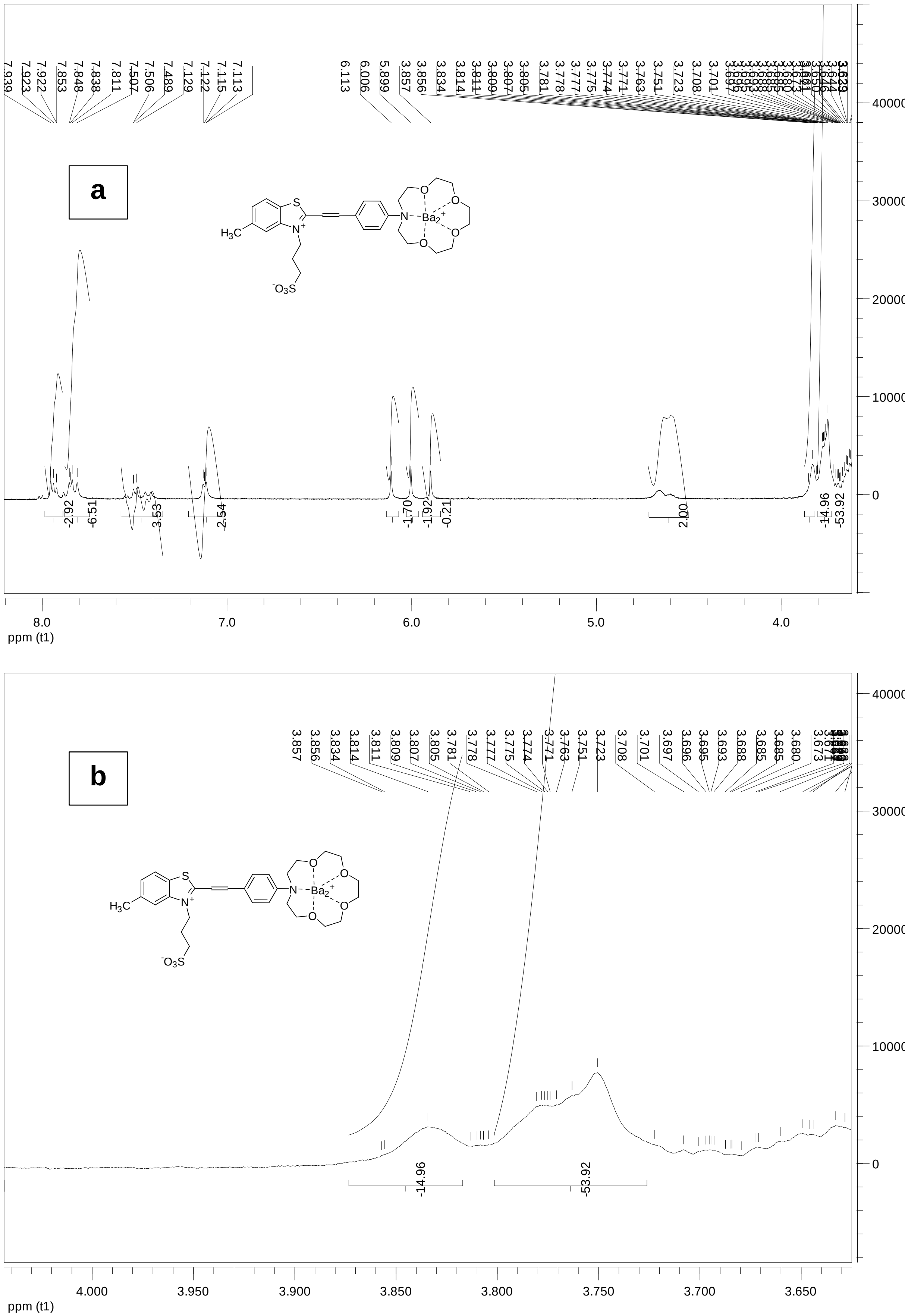

Figure S10: ^{1}H NMR spectra of Ba^{2+} (0.2 M) complex of compound **4b** (1×10^{-3} M in CD_3CN).

References

(1) Lednev, I. K.; Fyedorova, O. A.; Gromov, S. P.; Alfimov, M. V.; Moore, J. N.; Hester, R. E. *Spectrochimica Acta Part A: Molecular Spectroscopy*, **1993**, *49*, 1055-63.

(2) Lednev, I. K.; Hester, R. E.; Moore, J. N. *Journal of the Chemical Society, Faraday Transactions*, **1997**, *93*, 1551-8.

(3) Wu, C.-S.; Lin, Y.-J.; Chen, Y. *Organic & Biomolecular Chemistry*, **2014**, *12*, 1419-29.

(4) Alfimov, M. V.; Gromov, S. P.; Lednev, I. K. *Chemical Physics Letters*, **1991**, *185*, 455-60.
